# Subtype-specific conformational landscape of NMDA receptor gating

**DOI:** 10.1016/j.celrep.2024.114634

**Published:** 2024-08-17

**Authors:** Julia Bleier, Philipe Ribeiro Furtado de Mendonca, Chris H. Habrian, Cherise Stanley, Vojtech Vyklicky, Ehud Y. Isacoff

**Affiliations:** 1 Helen Wills Neuroscience Institute, University of California, Berkeley, Berkeley, CA 94720, USA; 2 Department of Molecular & Cell Biology, University of California, Berkeley, Berkeley, CA 94720, USA; 3 Biophysics Graduate Group, University of California, Berkeley, Berkeley, CA 94720, USA; 4 Weill Neurohub, University of California, Berkeley, Berkeley, CA 94720, USA; 5 Molecular Biology & Integrated Bioimaging Division, Lawrence Berkeley National Laboratory, Berkeley, CA 94720, USA; 6 Present address: Department of Molecular and Cellular Physiology, Stanford University School of Medicine, 279 Campus Drive, Stanford, CA 94305, USA; 7 Present address: DIANA Biotechnologies, a.s. Průmyslová 596, 252 50 Vestec, Czech Republic; 8 Lead contact

## Abstract

N-methyl-D-aspartate receptors are ionotropic glutamate receptors that mediate synaptic transmission and plasticity. Variable GluN2 subunits in diheterotetrameric receptors with identical GluN1 subunits set very different functional properties. To understand this diversity, we use single-molecule fluorescence resonance energy transfer (smFRET) to measure the conformations of the ligand binding domain and modulatory amino-terminal domain of the common GluN1 subunit in receptors with different GluN2 subunits. Our results demonstrate a strong influence of the GluN2 subunits on GluN1 rearrangements, both in non-agonized and partially agonized activation intermediates, which have been elusive to structural analysis, and in the fully liganded state. Chimeric analysis reveals structural determinants that contribute to these subtype differences. Our study provides a framework for understanding the conformational landscape that supports highly divergent levels of activity, desensitization, and agonist potency in receptors with different GluN2s and could open avenues for the development of subtype-specific modulators.

## In brief

Bleier et al. use single-molecule fluorescence resonance energy transfer (smFRET) in NMDA receptors to compare conformations and rearrangements of receptor subtypes with different GluN2 subunits. They find that low-open-probability receptors are more splayed and dynamic at rest, more compact when fully agonized, and show distinct subunit interactions.

## Graphical abstract

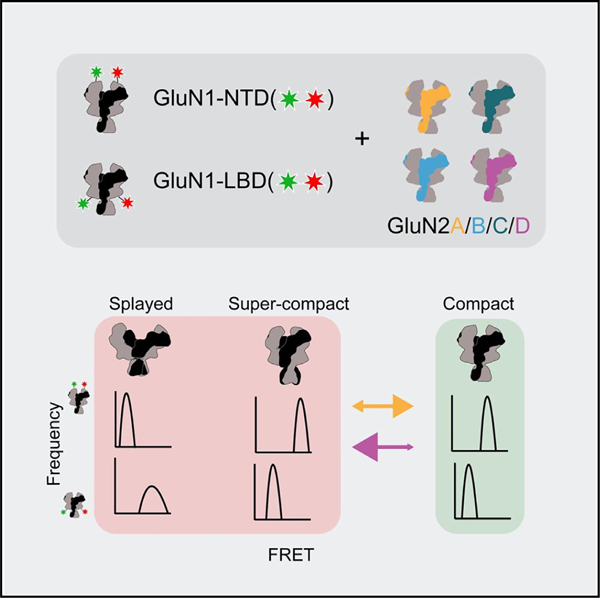

## INTRODUCTION

Fast glutamatergic neurotransmission mediated by ionotropic glutamate receptors (iGluRs) forms the basis of excitatory neurotransmission in the mammalian central nervous system. N-methyl-D-aspartate (NMDA) receptors (NMDARs) are unique among iGluRs in that they require both glutamate and glycine or D-serine in order to activate. Structurally, NMDARs are heterotetramers containing two pairs of alternating obligate GluN1 and variable GluN2 or GluN3 subunits, each consisting of an intracellular C-terminal domain (CTD), a pore-forming transmembrane domain, and a large extracellular domain made up of a ligand-binding domain (LBD) and an N-terminal domain (NTD), also known as the amino-terminal domain.^1,2^ Glutamate binding to the GluN2 LBD and glycine or D-serine binding to the GluN1 LBD are loosely coupled to channel opening,^3–5^ but even in saturating concentrations of agonists, the channel pore spends much of the time closed.^6–8^

Diverse synaptic signals can be transmitted by activating NMDA receptor subtypes with different GluN2 subunits, which exhibit several loosely covariant physiological properties as well as distinct spatiotemporal expression patterns, roles in synaptic transmission, plasticity, and disease.^9–13^ GluN2A NMDARs have the highest single-channel open probability (P_o_; ~50%), followed by GluN2B and GluN2C, with GluN2D having the lowest P_o_ (~1%).^9^ Subtypes with lower P_o_ exhibit less desensitization and slower deactivation^14–17^ and higher potency for both glutamate^18–21^ and glycine.^22,23^ In this way, low concentrations of glutamate, as seen in spillover from neighboring synapses, can activate low-P_o_ receptors with slow kinetics, and high-concentration glutamate transients in the synaptic cleft can activate higher-P_o_ receptors briefly in a precisely timed manner.

The conformational basis of these distinct properties has been the subject of intense study but remains incompletely understood. Kinetic analysis of single-channel studies has revealed that the activation pathways of NMDARs involve transit between multiple non-conducting closed and cation-permeable open states, which are thought to correspond to distinct conformations.^6,24–28^ The architecture and kinetics of these pathways differ significantly between receptor subtypes. Electrophysiological study of receptors with chimeric GluN2 subunits has also proven successful in identifying structural regions that contribute to observed properties.^21,23,29–31^ In particular, the NTD has been identified as a major contributor to subtype differences, including P_o_, sensitivity to allosteric modulators, deactivation rates, and agonist potency.

Structural studies have captured each diheteroterameric subtype at high resolution.^1,2,32–38^ Recent structures of fully agonist-bound receptors reveal that the degree of closure and twisting observed in the GluN2 NTDs roughly inversely correlates with receptor P_o_ across subtypes. Multiple classes of conformations have been observed in full agonists for most subtypes, which likely represent activation intermediates, but the open pore conformation has been elusive, consistent with its low stability. Important inroads into understanding active and inhibited conformations have been made in receptors with cross-linking mutations or negative allosteric modulators.^32,33,35,36,38^ However, it remains to be determined if these conformations are populated in the normal activation pathway. Moreover, gating properties cannot be understood without partially liganded and unliganded apo conditions, for which structures are largely missing or difficult to compare across subtypes due to differences in experimental conditions.^39^

The goal of the present study is to further our understanding of the conformational landscape that gives rise to NMDAR functional diversity. We sought to directly compare all diheteromeric subtypes under identical apo-like, partially, and fully liganded conditions. To do so, we used single-molecule fluorescence resonance energy transfer (smFRET), which has been used to study conformational pathways in ion channels and neurotransmitter receptors, including NMDARs.^40–45^ FRET acts as spectroscopic ruler, which allows us to directly examine non-conducting conformations that can only be deduced through kinetic modeling of electrophysiological recordings as well as to detect intermediates that may be too unstable for structural determination. Our experiments reveal stark differences in GluN1 conformation between receptors with different GluN2 subunits across apo-like, partially, and fully liganded conditions. Chimeric analysis identifies key extracellular domain components involved in these subtype differences.

## RESULTS

**Figure 1.**
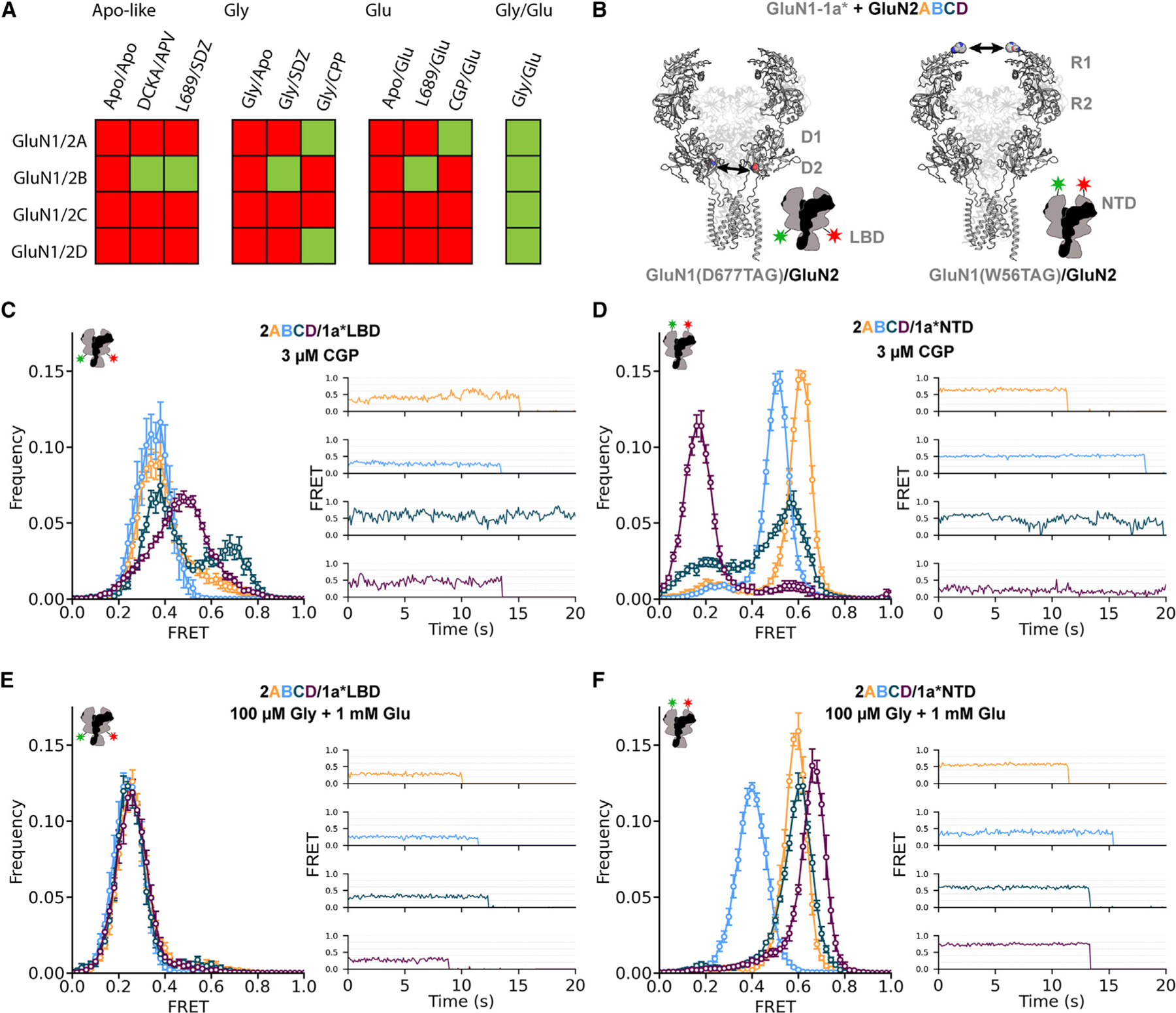
Agonists drive convergence of GluN1 conformation from GluN2-dependent resting FRET states (A) Analysis of existing structural data (red = 0 structures, green = 1 or more) for NMDA receptors GluN1/2A-D in apo-like conditions (GluN1 site/GluN2 site: apo/apo, 5,7-dichlorokynurenic acid [DCKA]/D-2-amino-5-phosphonovalerate [D-APV], L689,560/SDZ 220–040), partially liganded with glycine (GluN1 site/GluN2 site: glycine/apo, glycine/SDZ 220–040, glycine/CPP), partially liganded with glutamate (GluN1 site/GluN2 site: apo/glutamate, L689,560/glutamate, CGP78608/glutamate), and fully liganded (GluN1 site/GluN2 site: glycine/glutamate). (B) Spheres in structures (PDB: 7EOS ^36^) and stars in cartoons indicating labeling sites in D2 lobe of GluN1 (gray) LBD (D677) and R1 lobe of GluN1 NTD in NMDA receptors also containing GluN2 (transparent black in structure, black in cartoon). (C–F) Ligand-dependent conformations determined from intersubunit FRET between GluN1 LBDs with fluorophores incorporated at site D677TAG (C and E) or GluN1 NTDs with fluorophores incorporated at site W56TAG (D and F) in an apo-like condition (zero added glycine, 3 μM GluN1 antagonist CGP78608, zero added glutamate) (A and B) or with saturating concentrations of agonists (100 μM glycine, 1 mM glutamate) (C and D) when GluN1 is combined with GluN2A (orange), GluN2B (blue), GluN2C (teal), or GluN2D (magenta). (Left) Histograms of smFRET distributions indicating mean and SEM (error bars) across technical replicates of *n* = 4 movies or combined movies with total particle number across all movies listed in Table S1. (Right) Example smFRET traces with colors corresponding to histogram keys. Labeling sites indicated with green and red stars on cartoons. Donor (AF555) and acceptor (AF647) dyes were imaged at 10 fps.

Cryo-electron microscopy structures have been published for each of the four diheteroterameric GluN2 subtypes in the presence of both co-agonists, but only a few of the subtypes have been solved in approximations of partially liganded (only glycine or only glutamate) conditions, and only one with an approximation of the apo condition (Figure 1A). Additionally, it is difficult to compare across structures that use different antagonists, pair GluN2 subunits with different GluN1 splice variants, contain different stabilizing mutations or disulfide cross-links, and have been captured in different membrane environments and other experimental conditions. We sought to compare receptor conformation and dynamics across the four subtypes and ligands in identical conditions without any stabilizing mutations or truncations. We focused on measurement of two of the key components of the activation apparatus: the LBD and NTD. Given that GluN1 and GluN2 subunits interact extensively and that functional diversity emerges between receptor subtypes that differ in GluN2 but have the same GluN1, we decided to compare the structural dynamics of the common GluN1 subunit between receptors with each of the four different GluN2 subunits.

To attach the fluorophores site specifically, we incorporated an unnatural amino acid (UAA) that provides a bioorthogonal chemical handle to which the fluorophores could be conjugated in a chemically selective manner. We used an orthogonal pyrrolysyl-tRNA synthetase (pylRS) and tRNA to recode a TAG stop codon inserted within a protein to a supplied UAA, which carries a chemical handle.^46^ We transiently co-transfected three plasmids in HEK293T cells: one containing the pylRS and TAG-recognizing tRNA required for UAA incorporation, one containing the GluN1 subunit with an internal TAG stop codon at the desired probe placement site, and finally, a GluN2 subunit carrying a C-terminal human influenza hemagglutinin (HA) tag (Figure S1A). At the same time, we supplied the UAA, *trans*-Cyclooct-2-en-L-Lysine (TCOK), in the media. As the strained ring moiety in TCOK can react with tetrazine groups through click chemistry, incubating live cells with tetrazine containing fluorophores, pyrimidyl-tetrazine-AF555, and pyrimidyl-tetrazine-AF647 allowed us to stochastically label the TCOK-containing GluN1 subunits at specific sites.

Using this approach, ~50% of receptors contain one donor fluorophore and one acceptor fluorophore. Dual emission at the donor and acceptor wavelengths, single-step photobleaching, and dequenching of donor fluorescence upon acceptor photobleaching together confirm that our measurements come from single receptors. The remaining receptors contain either two donors or two acceptors, do not undergo FRET, and are not included in the analysis. Samples were labeled in live cells because the fluorophores do not cross the plasma membrane, ensuring that only cell-surface-exposed GluN1 subunits would be labeled. After labeling, we washed live cells to remove unreacted tetrazine dyes. We then lysed the cells and applied the lysate to a passivated glass coverslip sparsely presenting an anti-HA antibody, which allowed us to specifically pull down heterotetramers through the CTD of the unlabeled GluN2 subunits^42,47^ (Figure S1B). Once receptors were bound to the glass coverslip, we washed away unbound material, added ligands, and imaged bound receptors using total internal reflection fluorescence microscopy. The sparse distribution enabled us to resolve individual receptors (Figure S1C). By exciting the donor fluorophore of each receptor (in total internal reflection at 532 nm) and monitoring the emission of both its donor and acceptor, we calculated the FRET efficiency for each receptor (Figure S1D). Co-expressing different GluN2 subunits (GluN2A, GluN2B, GluN2C, or GluN2D) allowed us to directly compare conformational rearrangements of the same GluN1 subunit across each diheteromeric subtype. We tested several UAA incorporation sites and found reliable expression and function at GluN1 position W56 in the upper NTD and at D677 in the lower lobe of the LBD (Figures 1B and S1E).

We captured images at 10 fps, allowing us to follow slow conformational dynamics. Faster conformational changes (transitions between states corresponding to short-lived conformations with dwell times <100 ms), as seen in kinetic models from single-channel patch recordings, are not resolved at this speed of acquisition. As a result, observed FRET states represent a conformational ensemble. While structural studies provide all-atom high-resolution views of some of the classes that make up conformational ensembles, our smFRET approach has the potential to capture the occupancy distribution of the conformational ensemble and show how it changes under ligand conditions that have proved challenging for structural analysis. To compare our results with structural studies, we measured the distance between the two GluN1 W56 residues and the two D677 residues and converted them to the estimated FRET efficiency. For these estimates, we used the standard AF555/AF647 Förster radius (R_0_) of 51 Å, which is the distance between donor and acceptor fluorophores where energy transfer efficiency is 50% (FRET = 0.5) (Figure S2).^48^ For these estimates, we grouped studies using diverse antagonists, mutations, GluN1 splice variants, and other experimental conditions. The estimated FRET efficiency may differ from the measured FRET efficiency,^40^ but a direct internal comparison of relative FRET efficiencies for an individual FRET pair across GluN2 subtypes and ligand conditions is appropriate because we use one experimental setup with the same donor/acceptor pair incorporated at the same sites in the identical GluN1 subunits.

### Agonist-induced convergence of GluN1 LBD conformation

We first examined the conformation of the LBD in resting receptors without either co-agonist. We measured the distance between GluN1 subunits at position D677 in S2 of the lower LBD, proximal to the S2-M3 linker that couples the LBD to the channel gate (Figure 1B). Mimics of apo receptors, with both glycine and glutamate site antagonists, have been observed structurally but only in GluN2B receptors, where distinct antagonists and other conditions yielded divergent results (Figures 1A and S2).^35,49^ To observe apo-like receptors, we used the glycine LBD selective antagonist CGP78608 (CGP) in the absence of added glycine to compete out binding of trace glycine.^45^ With no glutamate or saturating (3 μM) CGP, we observe distinct conformational ensembles between the four GluN2 receptors (Figure 1C). The GluN2A receptor exhibits a major peak at a FRET efficiency of ~0.35 and a small tail at higher FRET. The GluN2B receptor shows a single large peak around at ~0.35. Like the GluN2A receptor, the GluN2C receptor has a major peak at ~0.35, though it has lower occupancy than in the GluN2A receptor. The GluN2C receptor also has a minor peak at ~0.7 with higher occupancy than the GluN2A receptor. The GluN2D receptor shows a broad peak centered at ~0.48, which extends from the low FRET peak to the high FRET peak of the others. Three-component Gaussian fits to the FRET distributions enable us to estimate relative occupancies of low (0.35), medium (0.50), and high (0.70) FRET lower-LBD states in the four GluN2 receptor subtypes (Figure S3). Occupancy of the lowest FRET state corresponding to the greatest separation between the lower LBDs followed the sequence GluN2B > GluN2A > GluN2C > GluN2D, demonstrating that the unliganded GluN2 subunit differentially affects GluN1 lower-LBD separation and stability in apo receptors.

We next turned to the study of the fully co-agonized receptor. In saturating glutamate (1 mM) and glycine (0.1 mM), we find that all four GluN2 receptors exhibit similar narrow lower-LBD FRET distributions centered at a peak of ~0.25 (Figure 1E). This is a lower FRET level than the lowest FRET component of the apo-like condition (at ~0.35). This difference is consistent with small changes in distance observed at this site with GluN1 LBD closure in GluN1b/GluN2B receptors between antagonist- and agonist-bound conformations (Figure S2).^35^ However, in other GluN2 subtypes, particularly GluN2C and GluN2D, a much larger reorientation to this low FRET state, which is expected to correspond to LBD-separated conformations, is observed. The similarity of the FRET between GluN1 LBD sites close to the M3 gate in fully agonized receptors across subtypes with such different P_o_ suggests that additional regions are important for determining P_o_.

### GluN2-dependent GluN1 NTD apo splaying and agonist compaction

Having observed large LBD conformational differences in the apo-like condition among the four GluN2 receptors but conformational similarity in the fully agonized condition, we turned to the GluN1 NTD, which allosterically modulates the activation pathway and so could differ between the subtypes in a manner that differentially regulates gating. We measured inter-GluN1 FRET between positions W56 in the R1 lobe of the NTD (Figure 1B). We find that the distribution of GluN1 NTD conformations in saturating glutamate and glycine differs greatly among the four GluN2 receptors. Each of the heterotetramers shows a dominant major FRET peak, with GluN2A at ~0.60, GluN2B at ~0.38, GluN2C at ~0.62, and GluN2D at ~0.70 (Figure 1F). These differences in NTD compaction are consistent with structural data where the estimated FRET between W56 sites in the dominant classes in full agonists follows the trend GluN2A ≲ GluN2C < GluN2D, where the degree of GluN1 compaction corresponds to increased closure and twisting of the GluN2 NTD clamshells (Figure S2).^33,36–38^ At least three structural classes of conformations have been observed in GluN2B receptors in the presence of glycine and glutamate, which may underlie the broader FRET distribution we observed (Figure S2).^32,35^

The shape of the FRET distributions also differs across subtypes, indicating a further difference in occupancy of discrete GluN1 NTD conformations. The GluN2A receptor exhibits the narrowest and most symmetric distribution, indicating relatively little conformational heterogeneity. In contrast, the other subtypes have broader distributions, and GluN2C and GluN2D are negatively skewed, reflecting some occupancy of less compact GluN1 NTD conformations. These less compact conformations may include the minor structural class observed in GluN2C receptors with lower estimated FRET, in which the NTDs are splayed open (Figure S2).^37,38^

In the apo-like condition with saturating CGP and no added glycine or glutamate, the low FRET component, which is minimally occupied in the presence of agonists in GluN2C and GluN2D receptors, is increasingly and differentially populated across subtypes (Figure 1D). GluN2A has a major FRET peak at ~0.60 and a small peak at ~0.20. GluN2B exhibits a major FRET peak at ~0.50 FRET and a similar small peak at ~0.20. GluN2C has a smaller and broader high FRET peak at ~0.56 and a larger peak at ~0.20. GluN2D has a large main FRET peak at ~0.18 and a minor peak at ~0.60. Thus, the occupancy of low FRET splayed states follows the sequence GluN2A ≅ GluN2B < GluN2C < GluN2D.

Taken together, GluN2 subunits distinctly influence inter-GluN1 NTD conformation in both the apo-like resting condition and saturating agonists as well as the inter-GluN1 LBD conformation in the resting condition. We note that in the resting condition, the NTD-NTD distance roughly mirrors the distance between lower LBDs, i.e., a greater distance (lower FRET) between NTDs corresponds to shorter distance and increased mobility (higher FRET and broader distributions) LBDs (compare Figures 1C and 1D), consistent with coordinated rearrangements of these domains. However, the shapes and relative shifts of the conformational distributions between the GluN1 LBD and NTD are not exact mirror images, showing that these rearrangements are not rigidly coupled. The degree of conformational change observed between apo-like and saturating agonist conditions also differs between subtypes. GluN2D receptors exhibit a particularly large conformational change between the dominant conformations of the resting condition, where the GluN1 NTDs are very far apart, and in the presence of saturating agonists, where the NTDs are closely opposed. In contrast, GluN2A receptors demonstrate very limited GluN1 NTD conformational changes between the apo-like and saturating agonist conditions. GluN2B and GluN2C receptors show intermediate behavior.

### GluN2 dependence of single-agonist GluN1 conformation

**Figure 2.**
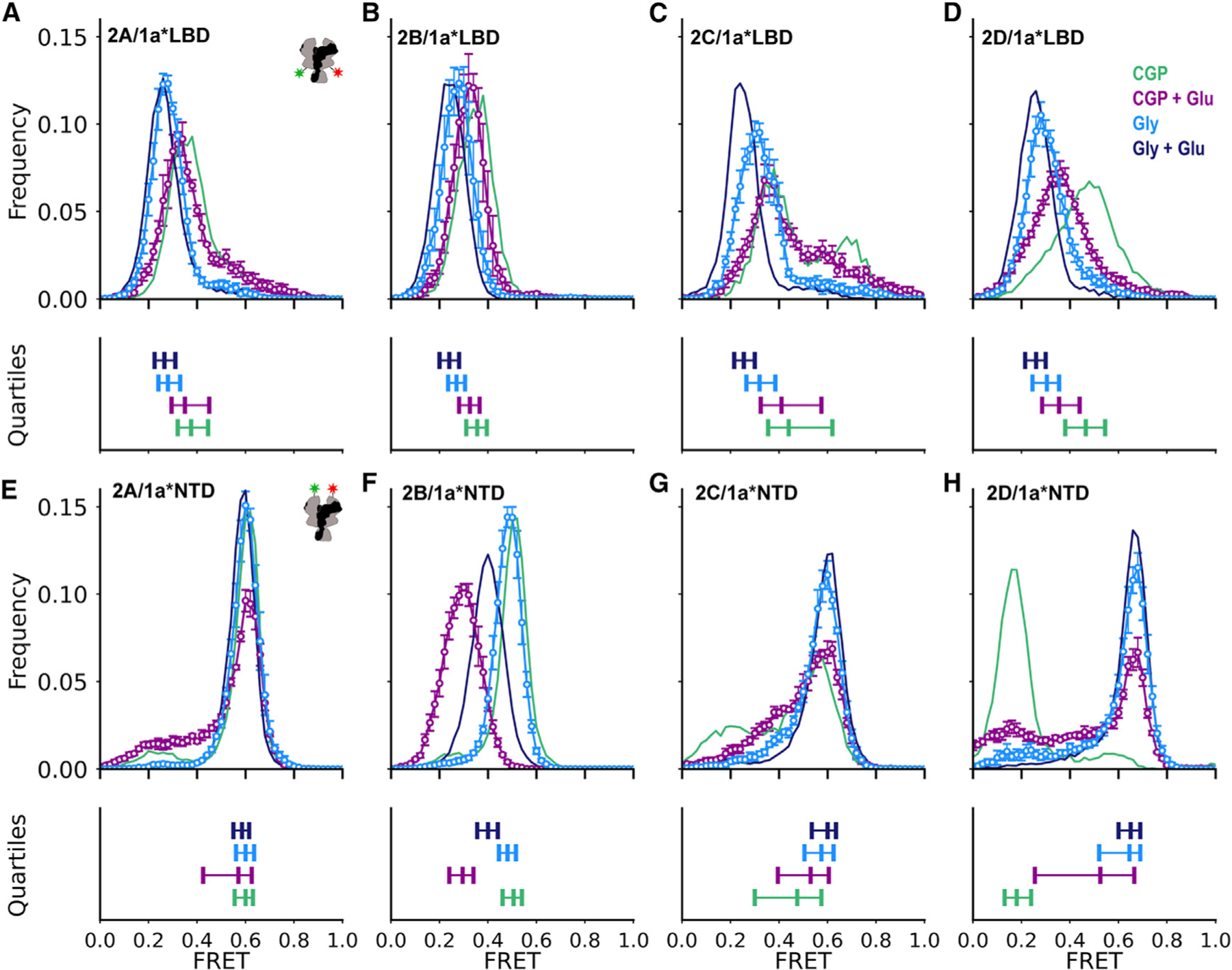
GluN2-dependent regulation of GluN1 conformation in single agonists Ligand-dependent conformations determined from intersubunit FRET between GluN1(D677TAG) lower LBD (A–D) or GluN1(W56TAG) NTD (E–H) paired with GluN2A (A and E), GluN2B (B and F), GluN2C (C and G), or GluN2D (D and H) in 3 μM CGP78608 and 1 mM glutamate (purple) or 100 μM glycine and zero added glutamate (blue) or in apo-like state (zero added glycine, 3 μM GluN1 antagonist CGP, zero added glutamate) (green) or saturating agonists (100 μM glycine, 1 mM glutamate) (navy) reproduced from Figure 1. (Top) Histograms of smFRET distributions indicating mean and SEM (error bars for those histograms not shown in Figure 1) across technical replicates of *n* = 4 movies or combined movies with total particle number across all movies listed in Table S1. Labeling sites indicated with green and red stars on cartoon insets (A and E). (Bottom) Quantification of the spread of the distributions in above histograms using the same color scheme. For each, the left and right vertical ticks indicate the first and third quartiles and the middle the median. Donor (AF555) and acceptor (AF647) dyes were imaged at 10 fps. See also Figures S3 and S4.

To understand the conformational activation pathway between resting and fully agonist-bound conditions, we assessed the behavior of GluN1 in partially liganded receptors. We asked how glycine-induced GluN1 LBD closure (saturated glycine, no glutamate) or glutamate-induced GluN2 LBD closure (CGP, no added glycine, saturating glutamate) affect inter-GluN1 lower-LBD distance in each of the diheterotetramers. In all subtypes, glycine produces a larger shift toward lower FRET, more separated conformations (Figures 2A–2D, cyan) than glutamate (Figures 2A–2D, purple). However, glutamate binding does have an allosteric influence on the conformation of the GluN1 subunit for all subtypes, shifting the FRET distribution in the direction of the low FRET distribution seen with full agonism (saturating glycine and glutamate). This shift is particularly large for GluN2D receptors, which occupy higher FRET states in the apo-like condition, though the broad distributions resulting from dynamics in individual traces remain.

In the GluN1 NTD, we observe a GluN2 dependence of both the degree and direction of shift in inter-NTD FRET efficiency in partially liganded conditions. In each of the GluN2 receptor subtypes, glycine drives receptors into the subtype-specific higher FRET state observed in fully agonized receptors (Figures 2E–2H, cyan). In GluN2A or GluN2B receptors, this shifts the small minority of receptors that are splayed in the apo-like condition, while in GluN2C and GluN2D receptors, it shifts the larger minority of splayed receptors (GluN2C) and the splayed majority (GluN2D). Saturating glutamate has a similar effect to glycine in GluN2C and GluN2D receptors, though to a lesser extent than glycine (Figures 2G and 2H, purple). In GluN2D receptors, the redistribution of occupancies in glutamate is due to frequent and heterogeneous transitions between several low and high FRET states (Figure S4). Strikingly, glutamate binding to GluN2A and GluN2B has the opposite effect on the GluN1 NTD compared to what is observed in GluN2C and GluN2D receptors: decreasing occupancy of the high FRET compact states (Figures 2E and 2F, purple). Additionally, while in glutamate, GluN2A, GluN2C, and GluN2D receptors occupy both compact and splayed FRET states, GluN2B receptors populate an intermediate FRET (~0.30) state (Figure 2F, purple), another property that sets the behavior of the GluN1 subunit of GluN2B receptors apart.

### Identification of structural determinants for GluN2 regulation of GluN1 conformation

**Figure 3.**
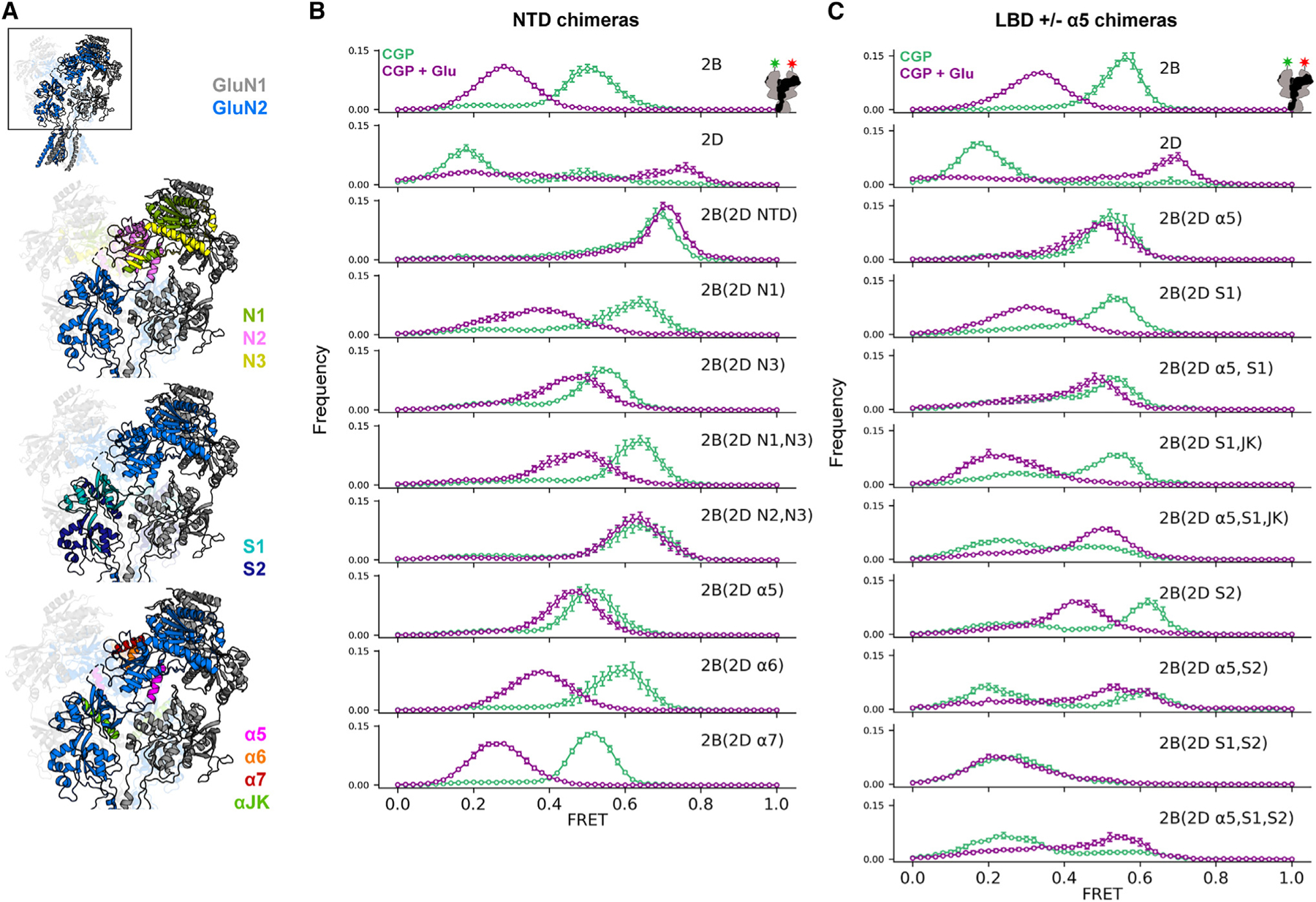
GluN2B/2D chimeras reveal structural determinants of subtype-specific NTD conformational dynamics (A) GluN1a/GluN2B glycine/glutamate structure (PDB: 7SAA ^61^) showing the regions swapped in chimeric GluN2 receptors. (B and C) Histograms of smFRET distributions indicating mean and SEM (error bars) across technical replicates of *n* = 4 individual movies or combined movies for ligand-dependent conformations determined from intersubunit FRET between GluN1(W56TAG) NTD paired with chimeric GluN2 subunits (according to scheme in Figure S5), which transplant pieces of GluN2D into GluN2B, focusing on the NTD (B) and LBD (C) in 3 μM CGP78608 and 1 mM glutamate (purple) and apo-like (zero added glycine, in 3 μM CGP78608; green) conditions. Labeling sites are indicated with green and red stars on cartoon insets at top right of (B) and (C). Total receptor number for each condition is listed in Table S1. Donor (AF555) and acceptor (AF647) dyes were imaged at 10 fps. See also Figure S5.

Having observed GluN2-dependent regulation of GluN1 conformation, we sought to understand the underlying allosteric mechanisms. In order to identify molecular determinants that mediate the influence of GluN2s on GluN1, we generated chimeras between GluN2B and GluN2D (Figures 3A and S5). We selected these two for their large differences in NTD conformation in the apo-like (CGP) condition, where the GluN1 NTD exhibits a dominant peak FRET efficiency at ~0.50 in the GluN2B receptor and ~0.18 in the GluN2D receptor, while in the glutamate-only (CGP+Glu) condition, there is a dominant peak at ~0.30 in the GluN2B receptor and at ~0.68 in the GluN2D receptor (Figure 3B, top two distributions).

We first swapped the GluN2D NTD into GluN2B (2B(2D NTD)) (Figure 3B). We observe that, like with GluN2D, a high FRET state is observed in glutamate, but a similarly high FRET state is observed in apo-like receptors. This suggests that the GluN2D NTD is sufficient for compaction in the glutamate condition but not for apo-like separation observed in GluN2D. To narrow down the critical region for regulation by glutamate, we split the NTD into three regions (N1–N3) and swapped the portion of GluN2D into GluN2B. GluN2B with the GluN2D N1 and N2 or N2 alone did not express well enough to assay. GluN2B with the GluN2D N2 and N3 (2B(2D N2, N3)) showed GluN1 NTD glutamate conformations closest to what was seen with transfer of the entire NTD (Figure 3B), suggesting that N2 plays an important role. We further subdivided N2 by substituting individually each of its three alpha helices (α5, α6, α7). Of the three helices, replacement of the GluN2B α5 helix with that of GluN2D produced the biggest shift to a higher FRET state in glutamate (Figure 3B). This suggested that, in N2, α5 contributes most strongly to the high FRET state observed with glutamate.

As the GluN2D NTD did not transfer the characteristic low FRET GluN2D apo-like resting condition, we next examined the LBD. Substitution of the GluN2D LBD into GluN2B (2B(2D S1, S2)) resulted in a low FRET state in CGP that resembled that of GluN2D (Figure 3C). However, with glutamate, low FRET was also observed, as in GluN2B receptors. Substitution of both the GluN2D LBD and α5 helix (2B(2D α5, S1, S2)) results in both a low FRET resting state and high FRET glutamate state, effectively switching the conformational pattern observed in GluN2B to that of GluN2D (Figure 3C). Subdivision of the LBD showed that GluN2D S1 and helices J and K of S2 (2B(2D S1, JK)), which make up the upper lobe of the LBD, can replicate the effect of the entire LBD but only when combined with the α5 helix of the NTD (2B(2D α5, S1, JK)) (Figure 3C). Thus, the chimeric analysis indicates the critical nature of the α5 helix in conformational rearrangements of GluN2B receptors.

### GluN2B conformational changes influence GluN1-NTD proximity through GluN1 loop 2

**Figure 4.**
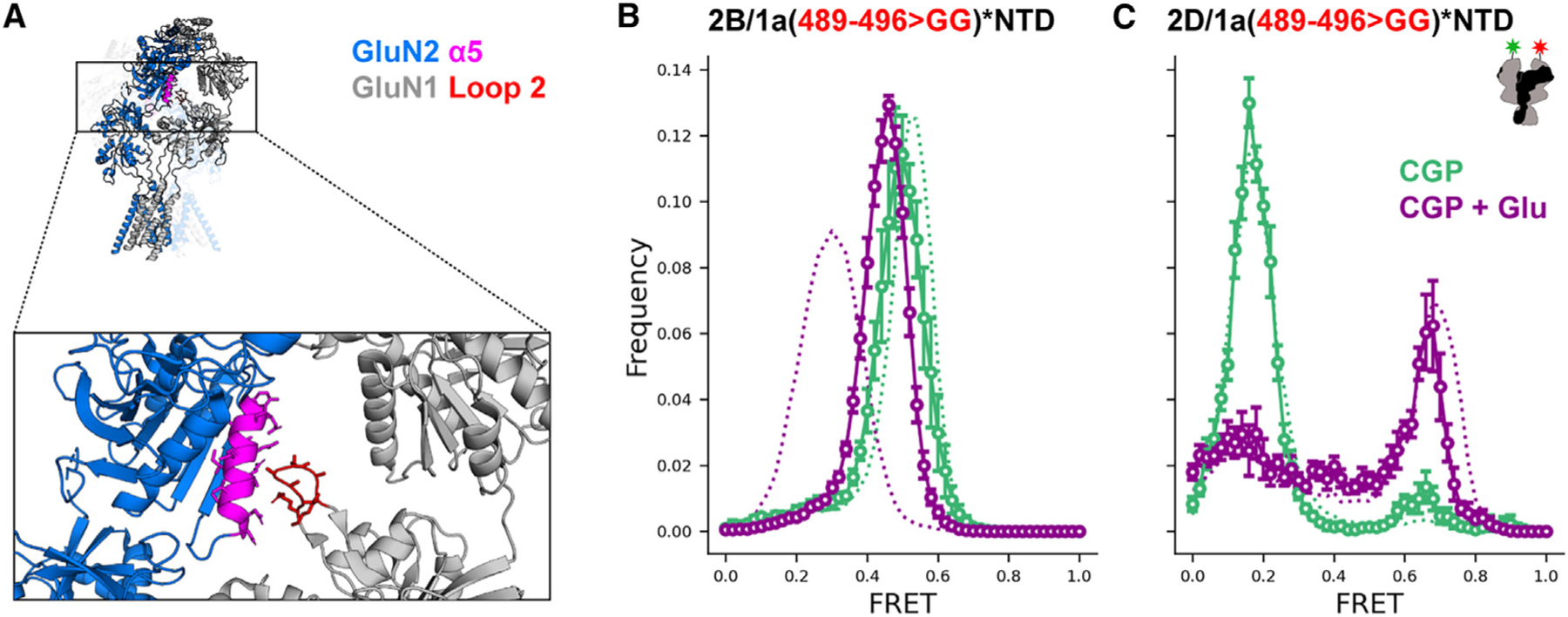
GluN1 loop 2 deletion affects glutamate-bound NTD conformation in GluN2B, but not GluN2D, receptors (A) GluN2 α5 helix (magenta) and residues 489–496 of GluN1 loop 2 (red) in the GluN1a/GluN2B glycine/glutamate structure (PDB: 7SAA ^61^). (B and C) Histograms of smFRET distributions indicating the mean and SEM (error bars) across technical replicates of *n* = 4 individual movies or combined movies for ligand-dependent conformations determined from intersubunit FRET between GluN1(W56TAG,R489-K496GG) (solid) or GluN1(W56TAG) (dotted) NTD paired with GluN2B (B) or GluN2D (C) in 3 μM CGP78608 and 1 mM glutamate (purple) and apo-like (zero added glycine, in 3 μM CGP78608) conditions. Labeling sites are indicated with green and red stars on cartoons. Total receptor number for each condition is listed in Table S1. Donor (AF555) and acceptor (AF647) dyes were imaged at 10 fps.

The GluN2B NTD α5 helix has been proposed to interact with GluN1 LBD loop 2 in an activation-state-dependent manner.^50^ We hypothesized that this interaction plays an allosteric role in linking glutamate binding to the inter-NTD GluN1 conformational changes observed in GluN2B receptors (Figure 4A). To test the role of this interaction, we mutated the 8 residue GluN1 loop 2 to a shorter 2 residue glycine linker. This disruption of GluN1 loop 2 maintained the FRET state observed in apo-like receptors but eliminated the separation of GluN1 NTDs that occurs in glutamate (Figure 4B), similar to what we observed with substitution of GluN2D α5 into GluN2B (Figure 3). However, pairing of GluN1 containing mutated loop 2 with GluN2D did not majorly affect the GluN1 NTD glutamate conformation in the GluN2D receptor (Figure 4C). These observations suggest that the interaction between GluN1 loop 2 and GluN2B α5 couples the GluN2B NTD to the GluN1 LBD and provides a mechanism through which glutamate binding to the GluN2 subunit allosterically regulates the conformation of the GluN1 subunit.

## DISCUSSION

We show that GluN2 subunits differentially determine the conformational trajectory from resting to fully agonized diheterotetrameric NMDARs. Single-channel studies use observations of transitions between closed and open states to construct models of the activation pathway and so, by definition, are carried out in the presence of both co-agonists. Similarly, a structural comparison of the four GluN2 receptor subtypes is currently available only with both co-agonists (Figure 1A). However, to understand the physiological response to agonists released at a synapse and the different properties of receptors composed of the different GluN2 subunits, it is also important to define the conformational pathway between unliganded and partially liganded conditions. smFRET allowed us to interrogate these conditions across the four diheterotetrameric subtypes and provide a view of key differences in conformational landscapes between subtypes with distinct properties.

In the ligand conditions assessed for which structures do exist, our FRET data generally correspond well with structural data. In glutamate and glycine together, the distance between W56 residues in the GluN1 NTDs is increased in structures of GluN2A receptors compared to GluN2D receptors (Table S2), which is expected to result in lower FRET in GluN2A receptors (Figure S2A), consistent with our results (Figure 1F). In GluN2B receptors, a wider range of distances is observed among the published structures, with the median distance being greater than that observed in GluN2A receptors (Table S2), resulting in lower predicted (Figure S2A) and observed (Figure 1F) FRET in GluN2B receptors. In GluN2C receptors, structural studies have observed a major class with proximal GluN1 NTDs, which would result in FRET values similar or slightly higher than those observed in GluN2A, as we observe with smFRET. The structurally observed minor class with separated GluN1 NTDs is not prevalent in our observations. The distance between D677 residues in the GluN1 LBDs is similar across structures of each receptor subtype in glutamate and glycine (Figure S2B), as we observe with smFRET (Figure 1E). In our smFRET data, we observe that glycine and glutamate each result in decreased FRET efficiency, corresponding to a greater distance between D677 residues in the GluN1 LBD. This generally holds true in conditions where structures are available, despite variable antagonists and cross-linking status (Figure S2B). A limited difference between glycine and glutamate conditions is observed in GluN2B receptors, where we observe closely overlapping FRET distributions (Figure 2B).

**Figure 5.**
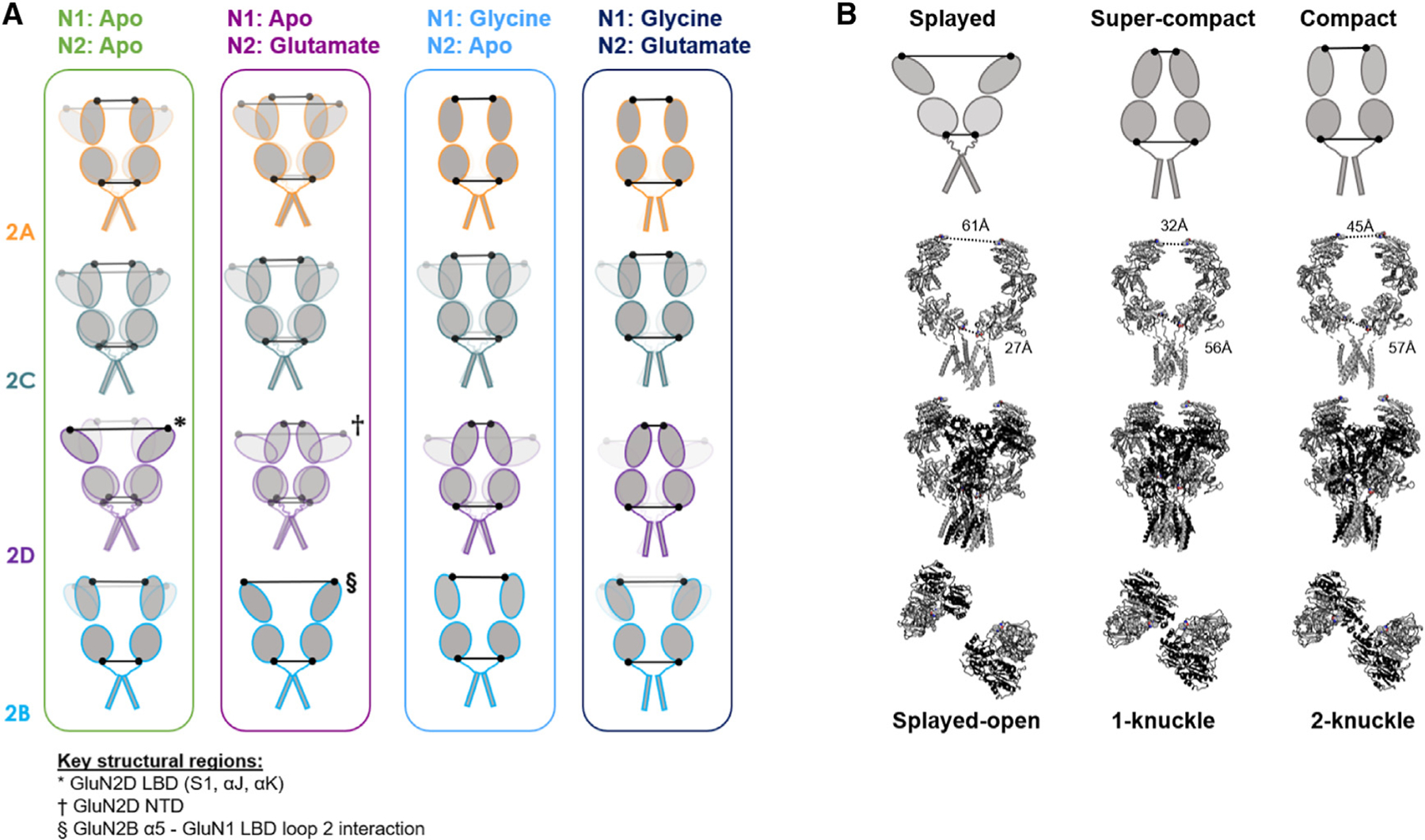
Subtype-specific conformational landscape of NMDA receptor gating (A) Cartoon representations of GluN1 subunits within NMDA receptor heterotetramers containing different GluN2 subunits (not depicted) based on smFRET distributions in ligand conditions representing steps in the activation pathway. Where multiple versions of a single domain are represented, opacity indicates relative occupancy. Black dots indicate locations of FRET probes and lines the distance between probes. Structural regions that were determined to be important for particular subtype-specific conformations are indicated, though they may not be the unique determinants. (B) Comparison of conformations deduced based on inter-GluN1 FRET and structurally observed conformations. (Top row) Cartoons of GluN1 in different conformations based on smFRET data. (Bottom three rows) Structures of GluN2A(black)/GluN1(gray) receptors^33^ in splayed-open (PDB: 6MMI; glycine/glutamate/1 μM ZnCl2 [pH 7.4]), 1-Knuckle (PDB: 6MM9; glycine/glutamate/1 mM ZnCl2 [pH 6.1]), and 2-Knuckle (PDB: 6MMP; glycine/glutamate/0.1 mM EDTA [pH 8.0]) conformations with W56 and D677 shown as spheres. Labeled lines indicate distances between β-carbons of GluN1 residues W56 in the NTD and D677 in the LBD. (Bottom row) Top-down view of the NTD showing (left to right) zero, one, and two alpha helices forming the tetrameric interface between GluN2 subunits.

Overall, our smFRET measurements show that GluN2A, GluN2C, and GluN2D receptors can cycle through a similar group of conformational ensembles, though with distinct distributions of occupancies (Figures 2E–2H). GluN2B receptors exhibit a different pattern of conformational changes at the NTD. A difference between GluN2A and GluN2B is expected based on prior work suggesting that they have distinct allosteric connections between the LBD and NTD layers.^35,36,51^ Our work demonstrates this distinct allostery in GluN2A and GluN2B receptors and suggests that GluN2C and GluN2D receptors have a similar allosteric route to GluN2A receptors (Figure 5A), despite their distinct energetics.

In a recent smFRET study, with donor and acceptor dyes attached to an N-terminal SNAP tag fused to GluN1, we observed in the GluN2B receptor a similar agonist-dependent pattern of conformations.^45^ We also observed the GluN1 NTD to undergo two steps of glutamate-induced separation, one with each glutamate binding event. Our observations here suggest that this glutamate-induced GluN1-NTD separation in the GluN2B receptor depends on the interaction between the GluN2B NTD α5 helix and loop 2 of the GluN1 LBD and that glutamate binding to the GluN2B LBD allosterically impacts the conformation of the GluN1 subunit through this interaction interface. This adds to earlier results suggesting that the transition between less and more active conformations involves the GluN1 LBD loop 2 switching from interacting with the upper to the lower region of the GluN2B NTD α5 helix.^32,35,50^

Among the conformationally similar GluN2A, GluN2C, and GluN2D receptors, the GluN2A receptor has been studied the most with structural approaches.^33,34,36^ In the presence of both co-agonists, it demonstrates a 2-Knuckle conformation (Figure 5B), in which two alpha helices of the GluN2A NTD participate in the tetrameric interface, W56 positions in the GluN1 NTDs come into a close-proximity “compact” conformation, and functionally, the channel opens with a high P_o_ (~0.5).^11^ In co-agonists plus the negative allosteric modulators zinc and protons, which induce closure of the GluN2A NTD clamshells and reduce P_o_, the distance between the R2 lobes of the GluN2A NTDs decreases, and the NTD interface assumes a 1-Knuckle conformation, involving only one GluN2A NTD alpha helix. With increased inhibition, the tetrameric interface falls apart, increasing the inter-NTD distance, including between GluN1 W56 positions, to assume “extended” and “splayed” conformations. In splayed conformations, the upper LBD D1-D1 interface is disrupted, and a proposed rocking motion of GluN2 LBDs brings D2 lobes together. This is expected to reduce tension on the linker to the channel gate and, thus, to reduce P_o_. Based on measurement of the distance between M3 helices, which form the main channel gate, and between the most proximal helices in the LBD, which may control tension on the gate, P_o_ is expected to be lowest in the 1-Knuckle and splayed conformations.

While in both co-agonists, the GluN2A receptor is found in a 2-Knuckle conformation, the GluN2D receptor occupies primarily 1-Knuckle-like conformations and GluN2C receptors in 1-Knuckle-like conformation with a minor class in a splayed-like conformation.^37,38^ Our inter-NTD measurements in full agonists reveal higher FRET between GluN1-NTDs in the GluN2D receptor than in GluN2A receptors (Figure 1), indicating greater proximity between GluN1 NTD R1 lobes, where W56 is located. This GluN2D receptor super-compact NTD conformation is expected to correspond to 1-Knuckle-like conformations and, therefore, low P_o_ (Figures 5 and S2). In GluN2C receptors, we observe primarily compact conformations with very low occupancy of low FRET splayed states. This difference could arise from the presence of the CTD in our study, as CTD truncation has large effects on channel activity.^52,53^ In full agonists, we observe little conformational difference in the GluN1 LBD lower lobe between receptor subtypes with very different gating patterns, suggesting that this GluN1 LBD activated conformation alone is insufficient for channel opening and that the differential bias on the gate between GluN2 receptor subtypes comes primarily from another rearrangement of GluN1 or GluN2.

In the apo-like condition, we find that subtypes with different GluN2 subunits adopt strikingly different conformational ensembles (Figure 1). GluN2A receptors, which exhibit the highest P_o_, adopt nearly the same ensemble of compact NTD conformations as in co-agonists, suggesting that they are poised in stable compact, 2-Knuckle-like conformations. The GluN1 LBD primarily occupies conformations that are similar to those adopted in full agonists in both GluN2A and GluN2B receptors. In lower P_o_ receptors, we observe decreased occupancy of poised compact NTD conformations in favor of partial and complete occupancy of splayed low FRET states in GluN2C and GluN2D receptors, respectively. At the same time, in GluN2C and GluN2D receptors, the GluN1 LBD undergoes a dynamic interchange between high and medium FRET states. This is consistent with a dynamic rocking LBD motion, similar to that proposed to occur in GluN2A LBDs in the splayed inhibited condition, upon rupture of the upper LBD interface.^33^ Indeed, our chimeric analysis independently reveals that the GluN2D upper LBD is key to setting the corresponding NTD splayed ensemble seen in resting receptors (Figure 3C). Though we have illustrated one example of a splayed conformation (Figure 5B), we expect that our measurements represent an ensemble of conformations that may differ from those observed structurally to date. These distinct resting conformational ensembles suggest that closure of the LBD clamshells in GluN2A receptors upon agonist binding may be sufficient to quickly open the channel gate, whereas opening of GluN2C and GluN2D receptors may be kinetically delayed until the upper LBD interface is reformed.

Partially liganded conformations are important to elucidate in order to understand activation kinetics, agonist cooperativity, and efficacy. In the GluN2A receptor, glycine alone and glutamate alone favor separation of the GluN1 lower LBDs, consistent with increased tension on the gate, but stabilize distinct NTD conformations, with glycine favoring high FRET, compact NTD states and glutamate favoring less compact, lower FRET NTD states (Figure 2), opposite effects that may reflect the known negative cooperativity between the co-agonists.^15,43,54–57^ In the GluN2 apo condition, glycine binding promotes the occupancy of conformations of the GluN1 agonized LBD and its NTD that are similar to those occupied with both co-agonists. This may explain why channel opening occurs more quickly when glutamate is applied in the background of glycine rather than the reverse in GluN2A receptors.^8^ GluN2D receptors exhibit high agonist potency for both glutamate and glycine compared to GluN2A receptors, which can largely be exchanged by exchanging their NTDs.^11,30^ As these measurements of potency are made in the presence of co-agonists, this suggests that the super-compact 1-Knuckle-like conformations observed in GluN2D receptors in glycine have higher affinity for glutamate than do the compact conformations observed in GluN2A receptors. Splayed conformations with more mobile GluN1 LBDs in the GluN2D receptor may have higher affinity for glycine than do the conformations observed in GluN2A receptors with more restricted LBDs. Similarly, in the structurally similar glutamate-gated AMPA receptors, desensitized conformations with ruptured upper LBD interfaces exhibit elevated glutamate affinity, potentially owing to decreased tension on the linker to the channel gate pulling open the LBD clamshell.^58–60^ The exact mechanisms through which these distinct conformations support distinct agonist affinity merit future study.

Our study reveals how each GluN2 subunit defines a distinct conformational landscape and allosterically influences GluN1 conformation along the NMDAR activation pathway. Our findings further our understanding of the conformational basis of subtype-specific receptor function and may be relevant for the design of subtype- and agonist-conformation-specific modulators.

### Limitations of the study

While our smFRET approach combines the dynamic readout on functioning receptors of single-channel patch-clamp recording with the use of FRET as a spectroscopic ruler, our analysis has several limitations. First, our readout is slow (10 fps), meaning that we miss out on fast transitions. An increase in camera frame rate would make it possible to follow slightly faster transitions, but the limited photon budget of each fluorophore would mean that we would have to sacrifice trace duration and would therefore miss out on slow transitions. Second, we measure conformations under steady-state ligand conditions and so miss the dynamics elicited by brief ligand pulses at synapses. This limitation is true too in most single-channel patch analysis. Third, our glycine-free condition uses the GluN1 antagonist CGP. We therefore refer to it as “apo-like.” We need to use CGP because the high affinity of the GluN1 agonist site means that even very low trace glycine in our solutions will result in some binding. We are confident in CGP as a good model for the apo receptor because we have shown that CGP acts as a neutral antagonist.^45^ Fourth, our point-to-point distance determinations only partially define receptor conformations. Expansion of FRET analysis to additional sites would enable better definition of the conformations. Fifth, our experiments are performed with receptors solubilized in detergent. Replacement of detergent with artificial bilayers, nanodiscs or vesicles—or even in cells—would better approximate the membrane environment of neurons, although the artificial bilayers do not entirely recapitulate the neuronal cell membrane, and in a live cell, the receptors would be free to diffuse, requiring tracking, which can lower the signal to noise of photon counting.

## STAR★METHODS

### RESOURCE AVAILABILITY

#### Lead contact

Requests for further information, resources, or reagents should be directed to and will be completed by the lead contact, Ehud Isacoff (ehud@berkeley.edu).

#### Materials availability

Plasmids generated in this study will be made available upon request.

#### Data and code availability

- All data reported in this paper will be shared by the lead contact upon request.
- This paper does not report significant original code.
- Any additional information required to reanalyze the data reported in this paper is available from the lead contact upon request.

### EXPERIMENTAL MODEL AND STUDY PARTICIPANT DETAILS

#### Cell culture and transfection

Human embryonic kidney 293T (ATCC: CRL-3216) were cultured in Opti-MEM (Gibco 31985070) supplemented with 5% fetal bovine serum on 3 μg/mL collagen coated plates at 37°C in 5% CO_2_. For smFRET experiments cells were cultured for approximately 3–25 passages before cells were seeded in 9.6cm^2^ 6-well plates coated with 0.5 mg/mL poly-L-lysine. At ~80% confluency, up to 6 wells of each construct combination were transfected. Media in each well was first replaced with 1 mL of Opti-MEM transfection media supplemented with 3% FBS, 20 mM MgCl2, 50 μM 5,7-dichlorokynurenic acid (5,7-DCKA), 800 μM D,L-2-amino-5-phosphonopentanoic acid (D,L-APV) and 20 μM ifenprodil. After 20 min, a mixture of 250 μL Opti-MEM, 5 μL lipofectamine and 5 μg of DNA (at a ratio of 5:1:5 of GluN1-1a plasmid with TAG stop codon at position W56 or D677; GluN2 subunit with a C-terminal GGGGSS linker followed by an HA tag (YPYDVPDYA); 4XpylT-EF1a-NES-Mm-PylRS(AF)-WPRE amber suppression plasmid). 12.5 μL of 25 mM *Trans*-cyclooctene lysine (TCOK) (SiChem) was added to each well for a final concentration of ~250 μM. TCOK stock was prepared at 100 mM in 0.2 M NaOH, 15% DMSO and was diluted 1:4 in 1 M HEPES before addition to cell media. Media was not changed again prior to receptor labeling on the day of imaging. For patch-clamp experiments the same procedure was followed with the following exceptions: cells were seeded on 18mm acid-washed borosilicate glass coverslips coated with poly-L-lysine (1 mg/mL) at a low density in 3.5cm^2^ 12-well plates; transfection occurred ~5 h later with transfection media consisting of 1.5% FBS, 20 mM MgCl2, 50 μM 5,7-DCKA and 400 μM D,L-APV. The mixture added after 20 min included 100 μL Opti-MEM, 2 μL lipofectamine and 1200 ng of DNA (500 ng GluN1-1a(W56TAG) or GluN1-1a(D677TAG), 100 ng GluN2 subunit with a C-terminal GGGGSS linker followed by an HA tag (YPYDVPDYA), 500 ng 4XpylT-EF1a-NES-Mm-PylRS(AF)-WPRE amber suppression plasmid, and 100 ng tdTomato).

### METHOD DETAILS

#### DNA constructs and site-directed mutagenesis

Amino acids and sites of mutations are numbered according to the wild type full length *rattus norvegicus* proteins (accession codes: BAA02498.1 (GluN2A), NP_036706.1 (GluN2B), XP_006247771.1 (GluN2C), NP_073634.2 (GluN2D)) beginning with methionine as 1. For GluN2 constructs, a flexible linker followed by a Human influenza hemagglutinin (HA) tag (GGGGS-YPYDVPDYA) was inserted immediately prior to the stop codon in the full-length protein. Chimeric GluN2 subunits (according to scheme in Figure S5 created using Boxshade^66^ with T-Coffee alignment^64^) involving large exchanged regions were generated using Gibson assembly and smaller regions and other modifications of GluN1 and GluN2 subunits were generated using PCR mutagenesis. Modifications in the Mm-PylRS-AF/Pyl-tRNACUA plasmid were generated using gBlocks (IDT) and Gibson assembly and included insertion of three additional copies of the pyrrolysyl-tRNA as well as insertion (the translation elongation factor EF1A and a nuclear export sequence MACPVPLQLPPLERLTLD from the HIV-1 transactivating protein Rev^67^) and removal (FLAG) of elements upstream and insertion of the woodchuck hepatitis virus post-transcriptional regulatory element^68^ downstream of the Pyrrolysyl-tRNA Synthetase(AF).

#### Patch-clamp electrophysiology

Patch-clamp recordings were performed 16–24 h following transfection. Each coverslip was washed in extracellular buffer (pH 7.4 with NaOH) containing, in mM: 160 NaCl, 2.5 KCl, 10 HEPES, 0.2 EDTA, 0.7 CaCl_2_, 1 MgCl_2_ and labeled in 300 nM of pyrimidyl-tetrazine-AF647 (JENA Biosciences) for 15–20 min. Following labeling, coverslips were transferred onto a recording chamber mounted on an Olympus IX71 inverted microscope, with a Mg^2+^-free version of the extracellular buffer additionally containing 100 μM glycine. Cells expressing TdTomato were identified using a DG-4 light excitation system (Sutter instruments). Voltage-clamp recordings were obtained using borosilicate glass pipettes with 4–6MΩ resistance filled with the following intracellular solution (in mM): 120 gluconic acid, 15 CsCl, 10 BAPTA, 10 HEPES, 3 MgCl_2_, 1 CaCl_2_, and 2 ATP-Mg salt (pH-adjusted to 7.2 with CsOH). After establishing whole-cell configuration, cells were held at −70mV. Liquid junction potential was not corrected. A second borosilicate glass pipette (2–4 MΩ) was loaded with 1 mM glutamate and positioned directly in front of the patched cell. A gentle positive pressure was applied to locally perfuse glutamate. Data was acquired using a CV203BU head stage, Axopatch 200B amplifier (Molecular Devices), and a Digidata 1440 acquisition board controlled with pCLAMP software, with data sampled at 10Khz, Bessel filtered at 4Khz.

#### NMDAR labeling and solubilization

Receptor labeling was performed on cells 24–48 h following transfection. Transfection media was removed and each well was washed twice in 1 mL extracellular buffer (pH 7.4 with NaOH) containing, in mM: 160 NaCl, 2.5 KCl, 2 CaCl_2_, 1 MgCl_2_, and 10 HEPES. 450 mL labeling solution containing 300 nM of each pyrimidyl-tetrazine-AF555 and pyrimidyl-tetrazine-AF647 (JENA Biosciences) in extracellular buffer was added to each well. The 6-well plate was then placed in an opaque container containing 4°C ddH2O and rocked gently at room temperature for 20 min. Following labeling, each well was washed with 1 mL extracellular buffer, 1 mL PBS (−/− Ca2+/Mg2+), and 1 mL PBS +1 mM PMSF (Thermo Scientific) was added. Cells were incubated at 4°C for ~5 min before being gently collected from the bottom of the well with a cell-scraper. Cell suspensions were spun down at 5000g for 5 min to pellet cells. Each pellet was resuspended in a lysis buffer containing (150 mM NaCl, 20 mM Tris (pH 8.0), 1% lauryl maltose neopentyl glycol (LMNG), 0.1% cholesteryl hemisuccinate (CHS) (Anatrace), protease inhibitor cocktail (Thermo Fisher Scientific), 1 mM PMSF, 5% glycerol) and allowed to shake in the dark for 90 min at 4°C. Following lysis, lysate was spun at 16,000g for 20 min and supernatant containing detergent-solubilized receptors was collected. This supernatant was subjected to 3 additional spins in 50 kDa Amicon Ultra 0.5 mL buffer exchange columns using a modified imaging buffer (pH 8.0 w/NaOH) consisting of (in mM) 160 NaCl, 2.5 KCl, 2 CaCl_2_, 10 MgCl_2_, 20 HEPES, and of 0.01% LMNG, 0.001% CHS to remove any remaining dye, inhibitors used in transfection, and glutamate.

#### NMDAR isolation and surface display

Imaging chambers for single-molecule experiments were prepared using aminosilane functionalized glass coverslips and slides. To prevent non-specific binding, slides were passivated with mPEG (Laysan Bio) and coverslips were passivated with mPEG and sparse biotin PEG{Citation}. 5–8 holes were drilled on each edge of a coverslip sized area of the slide prior to cleaning and passivation. Slides and coverslips were stored at −20°C until the day of each experiment. Double-sided adhesive was used to attach coverslips to slides and create several channels which were sealed with quick drying epoxy (Devcon) through which solutions could be flowed. On the day of each experiment, channels were incubated with 20 μg/mL NeutrAvidin (Thermo Fisher Scientific) for 15 min, followed by 1/100 biotinylated anti-HA antibody (Abcam, ab26228) for at least 1 hr. T50 buffer (50 mM NaCl, 10 mM Tris, pH 7.4) was used to dilute NeutrAvidin, anti-HA antibody as well as to wash each out of the chamber. Cell lysate was diluted (1–10x) and incubated in the imaging chamber (1–30 min) to achieve sparse mobilization. Unbound lysate was washed out extensively using a modified imaging buffer (pH 8.0 w/NaOH) consisting of (in mM) 160 NaCl, 2.5 KCl, 2 CaCl2, 10 MgCl2, 20 HEPES and of 0.01% LMNG, 0.001% CHS.

#### smFRET measurements

Receptors were imaged for smFRET in imaging buffer (pH 8.0 w/NaOH) consisting of (in mM) 160 NaCl, 2.5 KCl, 2 CaCl2, 10 MgCl2, 20 HEPES, 50 glucose, 0.01% LMNG, 0.001% CHS, 5 Trolox, and 2 protocatechuic acid. 50 nM protocatechuate-3,4-dioxygenase and any ligands were added into a total volume of 100 μL of imaging buffer immediately before it was loaded into imaging chamber. Micro-Manager 2.0.0-beta3^65^ was used to control excitation of donor fluorophores with a 532 nM laser (Cobolt) and acquisition with an objective-based TIRF microscope (1.65 NA, 60x Olympus) and Photometrics Prime 95B sCMOS camera at 100-ms frame rate. For each condition, at least 4 movies were collected in different regions of a single imaging channel. All experiments were repeated at least twice on separate days with similar results. Data included in individual figure panels was collected on the same day.

### QUANTIFICATION AND STATISTICAL ANALYSIS

#### smFRET analysis

smFRET data was processed using SPARTAN,^62^ where traces were extracted from acquired movies using the GetTraces module (with crosstalk = .15 and channel scaling of donor = 1, acceptor = 1.2), subjected to selection in AutoTraces (default criteria except FRET lifetime >50) and subsequent manual selection to ensure single-step bleaching of each fluorophore, constant total fluorescence, and global anti-correlation between donor and acceptors. From SPARTAN, display histograms (50 frames, bin size 0.02) and traces were exported (ForOrigin) and imported into a Jupyter notebook^63^ in which histograms of traces in individual movies (occasionally traces from up to 3 movies were combined in cases where expression was low) were averaged. Individual traces and population histograms showing mean of movies or combined movies were plotted. Error bars on population histograms correspond to the standard error of the mean (S.E.M.) across the sets of traces from individual movies. Median and quartiles were calculated as the FRET value corresponding to the first bin with over 25, 50 and 75% of cumulative counts for individual movies and averaged for each condition. Statistical parameters for each experiment are found in the figure legends. Trimodal Gaussian fitting was achieved using scipy.optimize.curve_fit with gaussians each defined as A*np.exp(-(x-mu_n)**2/(2*sigma**2)) with initial A = 0.05 and sigma = 0.01 and fixed means (mu_1 = 0.3, mu_2 = 0.5, mu_3 = .7). Distributions were not tested for normality prior to fitting. Area under each curve was calculated using Simpson’s rule (scipy.integrate.simpson) and reported as a percentage of the area under the trimodal fit.

## Supplementary Material

1

## Figures and Tables

**Table:** KEY RESOURCES TABLE

REAGENT or RESOURCE	SOURCE	IDENTIFIER
Antibodies		
Biotin Anti-HA tag antibody	Abcam	Cat# ab26228, RRID:AB_449023
Chemicals, peptides, and recombinant proteins		
NeutrAvidin Protein	Thermo Scientific	Cat# 31000
mPEG-SVA-5000	Laysan Bio	Item# MPEG-SVA-5000-1g
Biotin-PEG-SVA-5000	Laysan Bio	Item# Biotin-PEG-SVA-5000-1g
Protocatechuate 3,4-Dioxygenase	Sigma-Aldrich	Cat# P8279
Protocatechuic acid (3,4-Dihydroxybenzoic acid)	Tokyo Chemical Industry	Cat# C0055
CGP 78608	Tocris	Cat# 1493
DL-AP5	Tocris	Cat# 3693
Ifenprodil Hemitartrate	Tocris	Cat# 0545
5,7-Dichlorokynurenic acid	Tocris	Cat# 3698
Trolox (6-Hydroxy-2,5,7,8-tetramethylchroman-2-carboxylic Acid)	TCI America	Cat# H07261G
Pierce^™^ Protease Inhibitor Mini Tablets, EDTA-free	Thermo Scientific	Cat# A32955
trans-Cyclooctene-L-Lysine (TCO*A)	SiChem	Cat# SC-8008
Pyrimidyl-Tetrazine-AF555	Jena Bioscience	Cat# CLK-098
Pyrimidyl-Tetrazine-AF647	Jena Bioscience	Cat# CLK-102
Poly-L-Lysine	Sigma-Aldrich	Cat# P2636
Collagen I, Rat Tail	Gibco	Cat# A10483-01
Lauryl Maltose Neopentyl Glycol	Anatrace	Cat# NG310
Cholesteryl Hemisuccinate Tris Salt	Anatrace	Cat# CH210
Deposited data		
GluN1a/GluN2B with glycine/glutamate	Chou et al.^35^	PDB: 7SAA
GluN1/GluN2A with glycine/glutamate	Wang et al.^36^	PDB: 7EOS
GluN1/GluN2A with glycine/glutamate/1mMZnCl2/pH 7.4	Jalali-Yazdi et al.^33^	PDB: 6MMI
GluN1/GluN2A with glycine/glutamate/1uMZnCl2/pH 6.1	Jalali-Yazdi et al.^33^	PDB: 6MM9
GluN1/GluN2A with glycine/glutamate/.1mM EDTA/pH 8.0	Jalali-Yazdi et al.^33^	PDB: 6MMP
Experimental models: Cell lines		
Human: HEK293T cells	ATCC	CRL-3216
Recombinant DNA		
CMV-NR1-1a	Vyklicky et al.^45^	NP_058706.1
pcDNA3.1-GluN1-1a(W56TAG)	This paper	N/A
pcDNA3.1-GluN1-1a(D677TAG)	This study	N/A
CMV-GluN2A-HA	Vyklicky et al.^45^	BAA02498.1
CMV-GluN2B-HA	Vyklicky et al.^45^	NP_036706.1
CMV-GluN2C-HA	Monyer et al.^20^	XP_006247771.1
CMV-GluN2D-HA	Monyer et al.^69^	NP_073634.2
Mm-PylRS-AF/Pyl-tRNACUA	Spence et al.^46^	Addgene#122650
4XpylT, EF1a-NES-Mm-PylRS(AF)-WPRE	This paper	N/A
pcDNA3.1-GluN1-1a(W56TAG, R489-K496GG)	This paper	N/A
CMV-GluN2B-2D-NTD-HA	This paper	N/A
CMV-GluN2B-2D-N1-HA	This paper	N/A
CMV-GluN2B-2D-N3-HA	This paper	N/A
CMV-GluN2B-2D-N1-N3-HA	This paper	N/A
CMV-GluN2B-2D-N2-N3-HA	This paper	N/A
CMV-GluN2B-2D-α5-HA	This paper	N/A
CMV-GluN2B-2D-α6-HA	This paper	N/A
CMV-GluN2B-2D-α7-HA	This paper	N/A
CMV-GluN2B-2D-S1-HA	This paper	N/A
CMV-GluN2B-2D-α5,S1-HA	This paper	N/A
CMV-GluN2B-2D-S1,JK-HA	This paper	N/A
CMV-GluN2B-2D-α5,S1,JK-HA	This paper	N/A
CMV-GluN2B-2D-S2-HA	This paper	N/A
CMV-GluN2B-2D-α5,S2-HA	This paper	N/A
CMV-GluN2B-2D-S1,S2-HA	This paper	N/A
CMV-GluN2B-2D-α5,S1,S2-HA	This paper	N/A
Software and algorithms		
SPARTAN	Juette et al.^62^	https://www.scottcblanchardlab.com/software
PyMOL Molecular Graphics System, Version 2.0	Schrödinger, LLC	https://pymol.org/
Jupyter Notebook	Kluyver et al.^63^	https://jupyter.org/
T-Coffee	Notredame et al.^64^	https://tcoffee.crg.eu/apps/tcoffee/do:regular
Micro-Manager 2.0.0	Edelstein et al.^65^	https://micro-manager.org/Version_2.0
Boxshade	Albà^66^	https://junli.netlify.app/apps/boxshade/

## Highlights

- smFRET reveals conformational differences between NMDA receptors with different GluN2s
- Low-P_o_ receptors are more splayed at rest and more compact when fully agonized
- Interaction between the GluN1 LBD and GluN2 NTD contributes to these differences
