# Supplementary material for: Subtype-specific conformational landscape of NMDA receptor gating
Source: Cell Rep. Author manuscript; Available in PMC 2024 Oct 2. (PMC11446236; doi:10.1016/j.celrep.2024.114634)
Supplement: 1 [file NIHMS2019553-supplement-1.pdf]

Cell Reports, Volume *43*

Supplemental information

Subtype-specific conformational landscape of NMDA receptor gating

Julia Bleier, Philipe Ribeiro Furtado de Mendonca, Chris H. Habrian, Cherise Stanley, Vojtech Vyklicky, and Ehud Y. Isacoff

Supplemental Figures

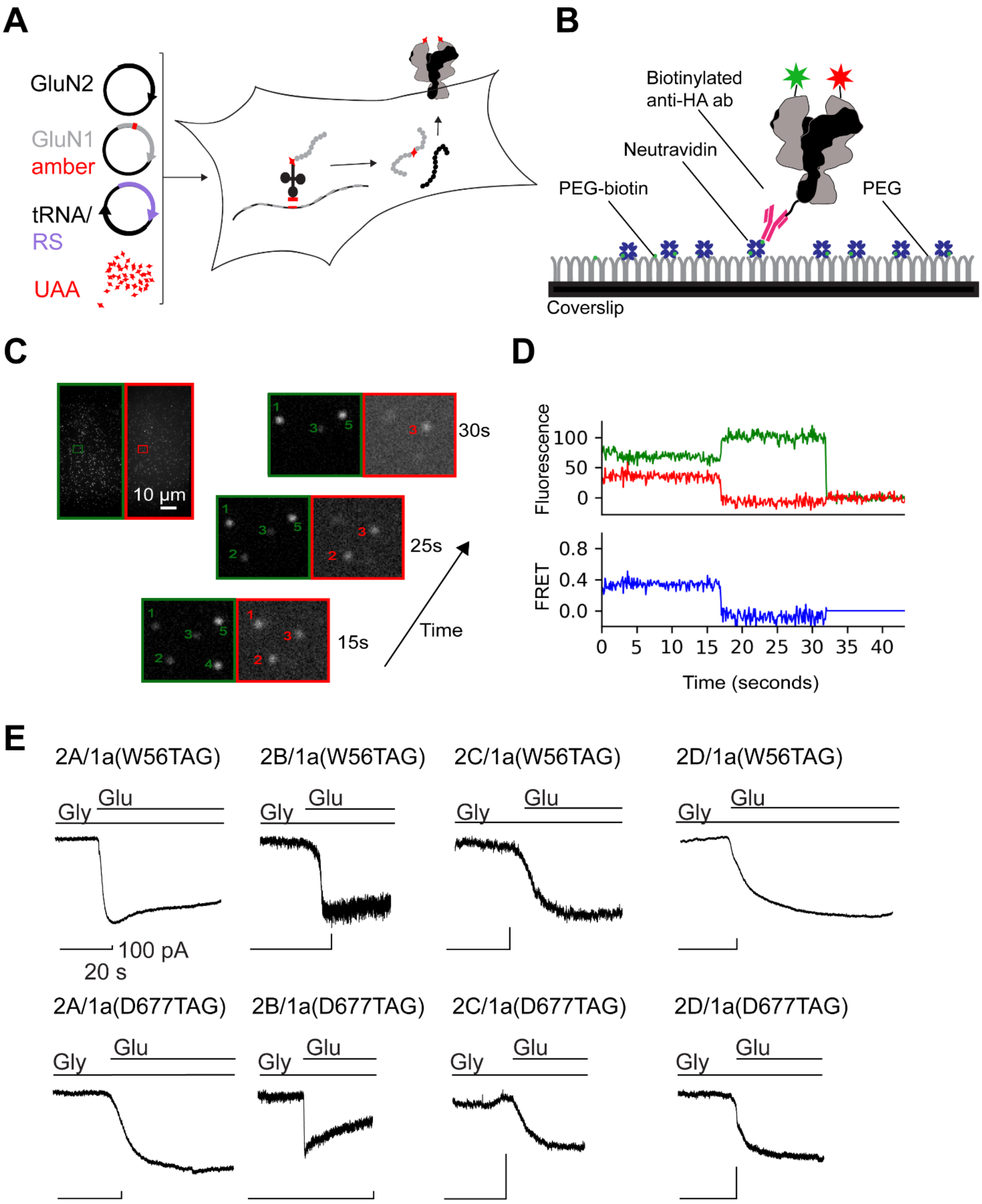

Figure S1. Expression and labeling of TCOK-incorporated NMDA receptors. Related to Figure 1 and STAR Methods. (A) Schematic of expression of NMDA receptors with site-specifically incorporated unnatural amino acids. Diagram inspired by Klippenstein et al. 2017[1]. (B) Schematic of labeled immune-purified NMDA receptor in imaging chamber with GluN2 HA tag bound to biotinylated anti-HA antibody, which is bound to neutravidin, which, in turn, is bound to biotin-PEG that adheres to the coverslip surface. Biotin-PEG is greatly outnumbered by unlabeled PEG, which passivates the coverslip to prevent non-specific protein binding. Thus, receptors are anchored at sufficiently low density for single receptors to be spatially resolved in imaging. (C) Total internal reflection excitation at 532 nm elicits donor fluorescence (green side) and acceptor fluorescence (red side) from single field of view split to two halves (~3400 square microns for each channel) of the CMOS sensor, showing entire image and expansion of small region with numbers indicating spots that contain donors (green) and acceptors (red). There are fewer spots visible in the acceptor channel because acceptor fluorophores are not directly excited by the 532 nm laser and are observed only through FRET from a donor. At 15 seconds, 5 donors and 3 acceptors are observed. Spots labeled 1, 2, and 3 contain both donor and acceptor, and the donor fluorescence is relatively dim due to FRET to the acceptor. Spots labeled 4 and 5, lack the acceptor and so are brighter, either because a single donor is not quenched by FRET to the acceptor, or because there are two unquenched donors. Only spots with both donor and acceptor are analyzed. After 25 seconds, the donor labeled 4 has photobleached as well as the acceptor labeled 1. Because FRET is no longer occurring, the donor labeled 1 is brighter at 25 seconds compared to at 15 seconds. This would result in a trace such as the example in panel D. At 30 seconds, no fluorescence is observed at spot 2. When the donor fluorophore photobleaches, FRET no longer occurs and fluorescence in the acceptor channel is no longer observed. Uniform contrast conditions which makes spots visible have been applied for the purpose of this visualization. (D) Example donor (green) and acceptor (red) fluorescence traces (top) and calculated FRET (blue) trace (bottom) under constant excitation at 532 nm over a 40 s time span from a single GluN1(D677TAG)/GluN2A receptor labeled on the LBD lower lobe of each GluN1 (UAA substituted at residue 677). The receptor is in the apo-like condition: zero agonist (no Gly, no Glu) and saturating GluN1 antagonist (3 μM CGP78608) to prevent binding by contaminating Gly. (This remedy needs to be taken for Gly, not Glu, because of the particularly high Gly affinity of the GluN1 LBD.) Initially, donor (upper trace, green) and acceptor (upper trace, red) fluorescence are stable, and their ratios indicate a FRET efficiency of ~0.4 (lower trace, blue). At ~17 s acceptor fluorescence abruptly drops to zero, a single-step (i.e. single molecule) photobleaching event that terminates FRET from the donor. At this time, the FRET trace drops to zero, and we observe simultaneous single-step donor dequenching to its full fluorescence level. At ~32 s the donor fluorescence abruptly drops to zero, also in a single step (i.e. single molecule) photobleaching event. Dual emission at the donor and acceptor wavelengths, single-step photobleaching, and dequenching of donor fluorescence upon acceptor photobleaching demonstrate that the measurements come from a single receptor labeled with a donor on one subunit and an acceptor on the other. Analysis is performed on traces from these receptors. To give equal analytic weight in histogram population analysis to each of the receptors that is analyzed, despite the fact that the stochastic nature of photobleaching means that some receptors bleach earlier than others, only the first 5 s of each trace are used in analysis. (E) Whole cell patch clamp current traces demonstrate that labeled GluN1(W56TAG)/GluN2A-D (top) or GluN1(D677TAG)/GluN2A-D (bottom) receptors in HEK293T cells retain functionality in response to pipette application of 1 mM glutamate in the presence of 100 μM glycine.

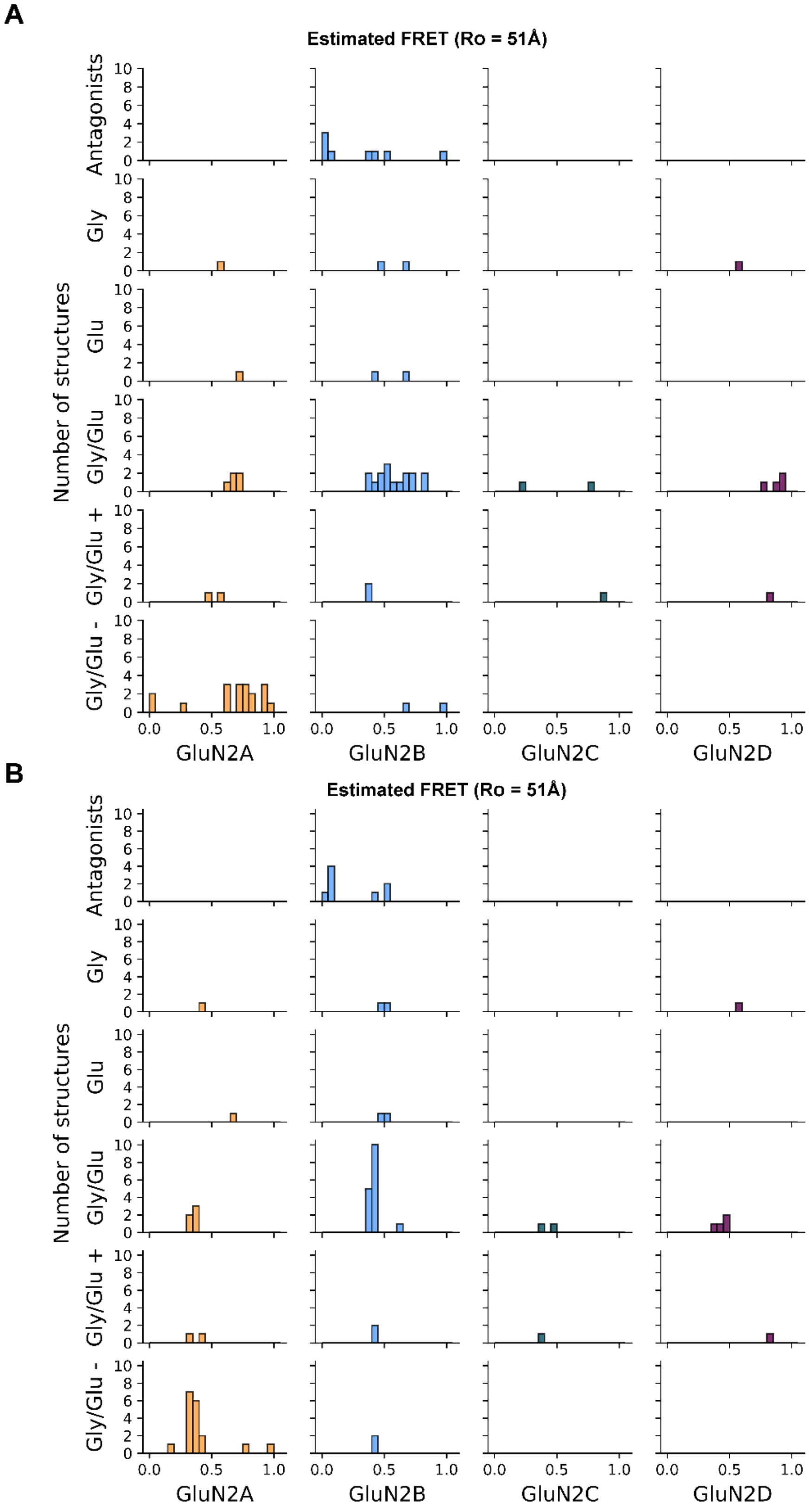

Figure S2. Estimated FRET from NMDAR structures. Related to Figures 1 and 2. For diheterotetrameric NMDAR structures in the PDB for which each extracellular domain was modeled distances (r) were measured between the β-Carbons of residues corresponding to the labeling sites (A) W56 and (B) D677 (or equivalents) in the two GluN1 subunits using PyMOL and converted to estimated FRET efficiencies with the formula FRET = $1/(1+(r/R_o)^6)$ with the Förster radius, R_o, = 51 based on standard parameters for the AF555/AF647 donor and acceptor pair.[2] For structures where a residue did not have a β-carbon in the model, the residue was mutated to itself in PyMOL before measurement. For structures where a residue needed for measurement was in a loop that was not modeled, the residues were added using the Builder in PyMOL and refined with ModLoop[3] prior to measurement to enable estimation. Structures were categorized based on their ligand condition (**Table S2**): Antagonists include structures with orthosteric antagonists of both GluN1 and GluN2. Gly includes structures with glycine and a GluN2 antagonist. Glu includes structures with a GluN1 antagonist and glutamate. Gly/Glu includes structures with glycine and glutamate. Gly/Glu+ (PAM) and Gly/Glu- (NAM) indicate modulators or other modifications which are associated with positive or negative allosteric modulation.

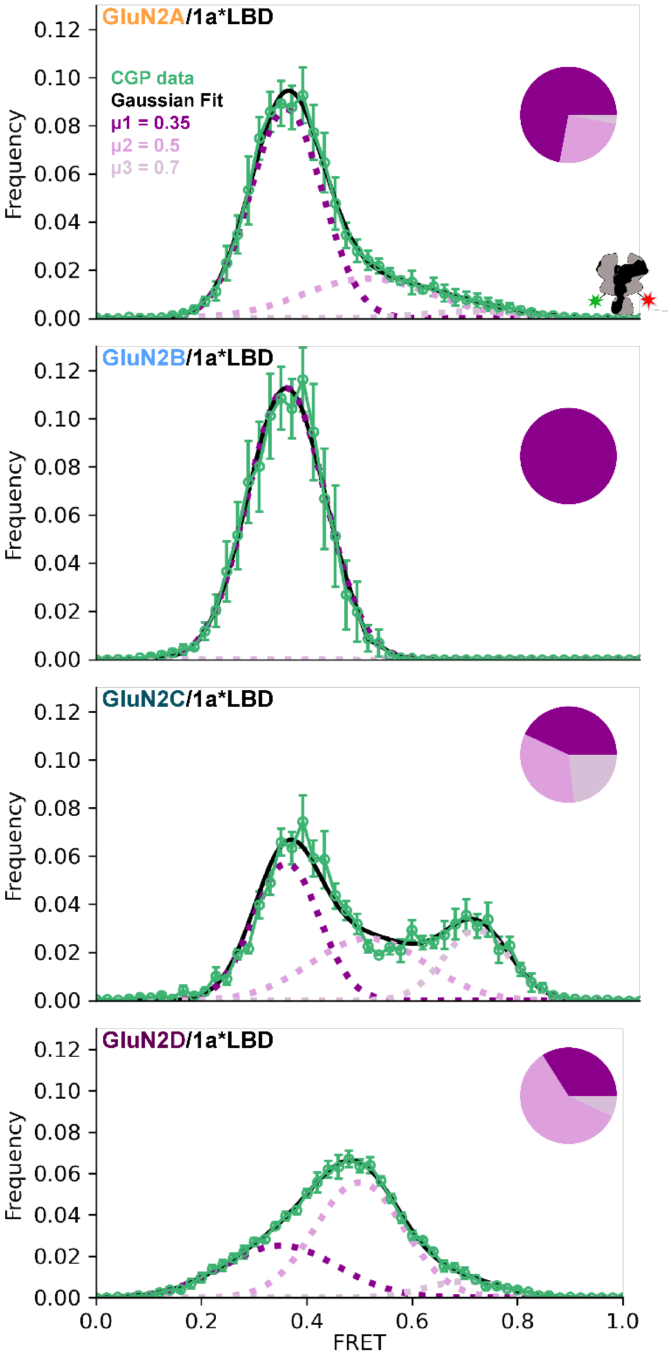

Figure S3. Gaussian fits. Related to Figure 1. Trimodal Gaussian fits (black) of inter-subunit FRET distribution (green, mean +/- S.E.M., from Fig. 1A) between GluN1(D677TAG) LBDs in Apo-like condition (zero added glycine, 3 μM GluN1 antagonist CGP78608, zero added glutamate) combined with each of the GluN2 subunits. Individual Gaussians centered at 0.35, 0.5, and 0.7 (dotted, shades of purple) with the corresponding percentages of area of the total fit in pie charts (right insets). Percentages, rounded to the nearest integer for Gaussians centered at FRET = 0.35: 72 (GluN2A), 100 (GluN2B), 43 (GluN2C), 34 (GluN2D); FRET = 0.5: 25 (GluN2A), 0 (GluN2B), 34 (GluN2C), 59 (GluN2D); FRET = 0.7: 3 (GluN2A), 0 (GluN2B), 23 (GluN2C), 7 (GluN2D).

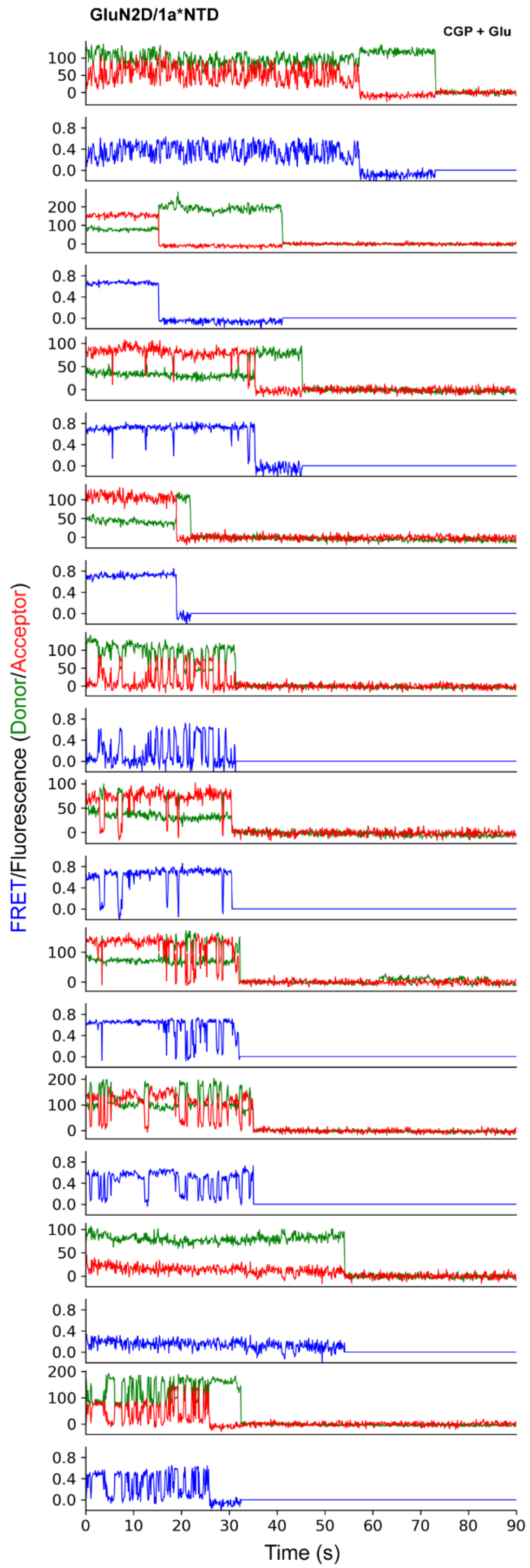

Figure S4. Example traces showing interconversions between splayed, compact and super-compact FRET states. Related to Figure 2. Reporting inter-subunit FRET (blue) between GluN1(W56TAG) NTD paired with GluN2D in 3 μM CGP78608 and 1 mM glutamate. These traces are representative of and included in the CGP + Glu histogram in Figure 2H. Donor (Alexa Fluor 555; green) and acceptor (Alexa Fluor 647; red) dyes imaged at 10 fps.

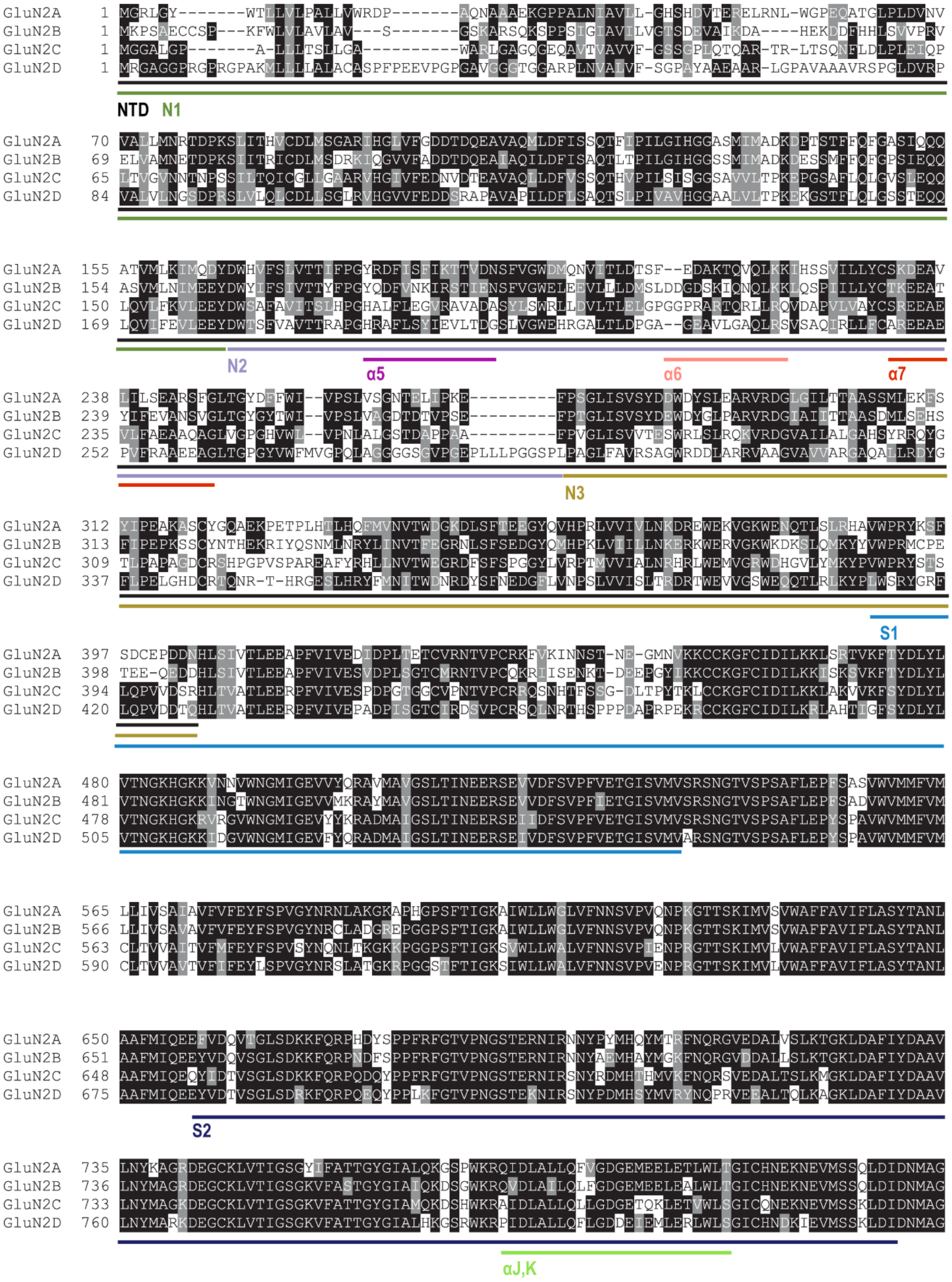

Figure S5. GluN2 sequence alignment. Related to Figure 3. Partial GluN2 sequence alignment (T-Coffee[4]) showing the regions that were exchanged in chimeric receptors, shaded with Boxshade.[5]

Table S1. Total particle number included in histograms for each condition, by figure.

Figure	Construct	Condition	Total # Particles Included
2	2B/1a(D677TAG)	3uMCGP + 1mM Glu	139
1,2,S3	2B/1a(D677TAG)	3uM CGP	101
1,2	2B/1a(D677TAG)	100uM Gly + 1mM Glu	226
2	2B/1a(D677TAG)	100uM Gly	243
2	2D/1a(D677TAG)	3uMCGP + 1mM Glu	172
1,2,S3	2D/1a(D677TAG)	3uM CGP	220
1,2	2D/1a(D677TAG)	100uM Gly + 1mM Glu	160
2	2D/1a(D677TAG)	100uM Gly	157
2	2A/1a(D677TAG)	3uMCGP + 1mM Glu	138
1,2,S3	2A/1a(D677TAG)	3uM CGP	157
1,2	2A/1a(D677TAG)	100uM Gly + 1mM Glu	116
2	2A/1a(D677TAG)	100uM Gly	246
2	2C/1a(D677TAG)	3uMCGP + 1mM Glu	88
1,2,S3	2C/1a(D677TAG)	3uM CGP	80
1,2	2C/1a(D677TAG)	100uM Gly + 1mM Glu	95
2	2C/1a(D677TAG)	100uM Gly	87
2	2B/1a(W56TAG)	3uMCGP + 1mM Glu	588
1,2	2B/1a(W56TAG)	3uM CGP	577
1,2	2B/1a(W56TAG)	100uM Gly + 1mM Glu	783
2	2B/1a(W56TAG)	100uM Gly	849
2,S4	2D/1a(W56TAG)	3uMCGP + 1mM Glu	266
1,2	2D/1a(W56TAG)	3uM CGP	116
1,2	2D/1a(W56TAG)	100uM Gly + 1mM Glu	290
2	2D/1a(W56TAG)	100uM Gly	229
2	2A/1a(W56TAG)	3uMCGP + 1mM Glu	425
1,2	2A/1a(W56TAG)	3uM CGP	452
1,2	2A/1a(W56TAG)	100uM Gly + 1mM Glu	330
2	2A/1a(W56TAG)	100uM Gly	416
2	2C/1a(W56TAG)	3uMCGP + 1mM Glu	236
1,2	2C/1a(W56TAG)	3uM CGP	276
1,2	2C/1a(W56TAG)	100uM Gly + 1mM Glu	355
2	2C/1a(W56TAG)	100uM Gly	335
3	2B(2D N1)/1a(W56TAG)	3uMCGP + 1mM Glu	206
3	2B(2D N1)/1a(W56TAG)	3uM CGP	215
3	2B(2D N3)/1a(W56TAG)	3uMCGP + 1mM Glu	608
3	2B(2D N3)/1a(W56TAG)	3uM CGP	487
3	2B(2D N1 N3)/1a(W56TAG)	3uMCGP + 1mM Glu	440
3	2B(2D N1 N3)/1a(W56TAG)	3uM CGP	186
3	2B(2D N2 N3)/1a(W56TAG)	3uMCGP + 1mM Glu	312
3	2B(2D N2 N3)/1a(W56TAG)	3uM CGP	373
3	2B/1a(W56TAG)	3uMCGP + 1mM Glu	812
3	2B/1a(W56TAG)	3uM CGP	566
3	2D/1a(W56TAG)	3uMCGP + 1mM Glu	322
3	2D/1a(W56TAG)	3uM CGP	184
3	2B(2D alpha5)/1a(W56TAG)	3uMCGP + 1mM Glu	641
3	2B(2D alpha5)/1a(W56TAG)	3uM CGP	487
3	2B(2D NTD)/1a(W56TAG)	3uMCGP + 1mM Glu	190
3	2B(2D NTD)/1a(W56TAG)	3uM CGP	182
3	2B(2D alpha6)/1a(W56TAG)	3uMCGP + 1mM Glu	671
3	2B(2D alpha6)/1a(W56TAG)	3uM CGP	548

3	2B(2D alpha7)/1a(W56TAG)	3uMCGP + 1mM Glu	592
3	2B(2D alpha7)/1a(W56TAG)	3uM CGP	617
3	2B/1a(W56TAG)	3uMCGP + 1mM Glu	317
3	2B/1a(W56TAG)	3uM CGP	270
3	2D/1a(W56TAG)	3uMCGP + 1mM Glu	169
3	2D/1a(W56TAG)	3uM CGP	145
3	2B(2D S2)/1a(W56TAG)	3uMCGP + 1mM Glu	192
3	2B(2D S2)/1a(W56TAG)	3uM CGP	156
3	2B(2D S1)/1a(W56TAG)	3uMCGP + 1mM Glu	408
3	2B(2D S1)/1a(W56TAG)	3uM CGP	299
3	2B(2D alpha5)/1a(W56TAG)	3uMCGP + 1mM Glu	124
3	2B(2D alpha5)/1a(W56TAG)	3uM CGP	150
3	2B(2D S1 S2)/1a(W56TAG)	3uMCGP + 1mM Glu	207
3	2B(2D S1 S2)/1a(W56TAG)	3uM CGP	270
3	2B(2D alpha 5 S1 S2)/1a(W56TAG)	3uMCGP + 1mM Glu	181
3	2B(2D alpha 5 S1 S2)/1a(W56TAG)	3uM CGP	210
3	2B(2Dalpha5 S1 JK)/1a(W56TAG)	3uMCGP + 1mM Glu	351
3	2B(2Dalpha5 S1 JK)/1a(W56TAG)	3uM CGP	342
3	2B(2Dalpha5 S1)/1a(W56TAG)	3uMCGP + 1mM Glu	314
3	2B(2Dalpha5 S1)/1a(W56TAG)	3uM CGP	244
3	2B(2Dalpha5 S2)/1a(W56TAG)	3uMCGP + 1mM Glu	108
3	2B(2Dalpha5 S2)/1a(W56TAG)	3uM CGP	107
4	2B/1a(W56TAG,489-496GG)	3uMCGP + 1mM Glu	684
4	2B/1a(W56TAG,489-496GG)	3uM CGP	563
4	2B/1a(W56TAG)	3uMCGP + 1mM Glu	348
4	2B/1a(W56TAG)	3uM CGP	380
4	2D/1a(W56TAG)	3uMCGP + 1mM Glu	119
4	2D/1a(W56TAG)	3uM CGP	151
4	2D/1a(W56TAG,489-496GG)	3uMCGP + 1mM Glu	107
4	2D/1a(W56TAG,489-496GG)	3uM CGP	97

Table S2. Structures analyzed in Figure S2.

PDB ID	GluN2	Classification	Inter-GluN1(W56) Distance (Å)	Inter-GluN1(D677) Distance (Å)
7EOS[7]	2A	Glycine+Glutamate+PAM	51.8	54.5
6IRA[8]	2A	Glycine+Glutamate	47.6	54.6
6MMG[6]	2A	Glycine+Glutamate	43.9	56.8
6MMP[6]	2A	Glycine+Glutamate	44.6	56.9
7EOR[7]	2A	Glycine+Glutamate+PAM	49	56.9
7EOT[7]	2A	Glutamate	43.3	45.8
7EOQ[7]	2A	Glycine	48.5	54
6IRF[8]	2A	Glycine+Glutamate+NAM	47.6	54.2
6IRG[8]	2A	Glycine+Glutamate+NAM	47.6	54.4
6IRH[8]	2A	Glycine+Glutamate+NAM	47.5	54.6
6MMA[6]	2A	Glycine+Glutamate+NAM	40.4	55.3
6MMB[6]	2A	Glycine+Glutamate+NAM	90.8	65.7
6MM9[6]	2A	Glycine+Glutamate+NAM	31.6	55.5
6MMH[6]	2A	Glycine+Glutamate+NAM	33	56.2
6MMM[6]	2A	Glycine+Glutamate+NAM	43.2	57.1
6MMI[6]	2A	Glycine+Glutamate+NAM	60.8	26.8
6MMJ[6]	2A	Glycine+Glutamate+NAM	112.3	41.7
6MMK[6]	2A	Glycine+Glutamate+NAM	35.2	56
6MML[6]	2A	Glycine+Glutamate+NAM	42.2	56.9
6MMR[6]	2A	Glycine+Glutamate	45.1	55.1
6MMN[6]	2A	Glycine+Glutamate+NAM	43.8	57.4
6MMS[6]	2A	Glycine+Glutamate	43.8	55.7
6MMT[6]	2A	Glycine+Glutamate+NAM	30.9	57.1
6MMU[6]	2A	Glycine+Glutamate+NAM	39.6	56.7
6MMV[6]	2A	Glycine+Glutamate+NAM	40.6	56.2
6MMW[6]	2A	Glycine+Glutamate+NAM	43.3	56.8
6MMX[6]	2A	Glycine+Glutamate+NAM	42.3	56.6
5FXG[9]	2B	Glycine+Glutamate	40.1	46.5
6WHT[10]	2B	Glycine+Glutamate	51.6	54.9
6WI1[10]	2B	Glycine+Glutamate+PAM	55.9	52.8
7TEQ[11]	2B	Glycine+Glutamate+PAM	55.6	53.3
5FXH[9]	2B	Glycine+Glutamate	50.3	54.2
5FXI[9]	2B	Glycine+Glutamate	54.3	54.3
5FXJ[9]	2B	Glycine+Glutamate	50.7	54.5
5FXK[9]	2B	Glycine+Glutamate	46.1	53.3
5IOU[12]	2B	Glycine+Glutamate	56	53.8
6WHS[10]	2B	Glycine+Glutamate	45.3	54.2
6WHR[10]	2B	Glycine+Glutamate	55.2	54.7

7SAA[13]	2B	Glycine+Glutamate	45.4	54.7
7TE9[11]	2B	Glycine+Glutamate	43	53.4
7TEB[11]	2B	Glycine+Glutamate	50.3	53.7
7TEE[11]	2B	Glycine+Glutamate	49.3	53.6
7TER[11]	2B	Glycine+Glutamate	51.8	54.7
7TES[11]	2B	Glycine+Glutamate	40.2	53.5
7TET[11]	2B	Glycine+Glutamate	44.2	54.8
5IPV[12]	2B	Antagonists	52.9	53.5
5IPQ[12]	2B	Antagonists	25.5	80.3
5IPR[12]	2B	Antagonists	78.2	83.6
5IPS[12]	2B	Antagonists	92.9	80.8
5IPT[12]	2B	Antagonists	124.2	81.1
5IPU[12]	2B	Antagonists	147.8	81.9
6WHW[10]	2B	Glycine	52.2	52.7
6WHX[10]	2B	Glycine	45.9	51
6WHY[10]	2B	Glutamate	52.9	51
6WI0[10]	2B	Glutamate	45.2	52.3
6WHU[10]	2B	Antagonists	56.1	50.4
6WHV[10]	2B	Antagonists	50	49.8
4PE5[14]	2B	Glycine+Glutamate+NAM	30.1	54.3
5IOV[12]	2B	Glycine+Glutamate+NAM	44.5	53.5
7YFG[15]	2C	Glycine+Glutamate	40.9	54.8
7YFH[15]	2C	Glycine+Glutamate+PAM	36.9	54.6
8HDK[15]	2C	Glycine+Glutamate	63.6	52.4
8E96[16]	2D	Glycine+Glutamate	36.7	53.1
7YFF[15]	2D	Glycine	47.9	49.2
7YFL[15]	2D	Glycine+Glutamate	32.4	55
7YFM[15]	2D	Glycine+Glutamate	35.2	51.7
7YFO[15]	2D	Glycine+Glutamate+PAM	38.4	39.4
7YFR[15]	2D	Glycine+Glutamate	42.3	51.7

References

1. Klippenstein, V., Hoppmann, C., Ye, S., Wang, L., and Paoletti, P. (2017). Optocontrol of glutamate receptor activity by single side-chain photoisomerization. eLife *6*, e25808. 10.7554/eLife.25808.

2. Lambert, T.J. (2023). Using FPbase: The Fluorescent Protein Database. In Fluorescent Proteins: Methods and Protocols, M. Sharma, ed. (Springer US), pp. 1–45. 10.1007/978-1-0716-2667-2_1.

3. Fiser, A., and Sali, A. (2003). ModLoop: automated modeling of loops in protein structures. Bioinforma. Oxf. Engl. *19*, 2500–2501. 10.1093/bioinformatics/btg362.

4. Notredame, C., Higgins, D.G., and Heringa, J. (2000). T-Coffee: A novel method for fast and accurate multiple sequence alignment. J. Mol. Biol. *302*, 205–217. 10.1006/jmbi.2000.4042.

5. Albà, M. (2000). Making alignments prettier. Genome Biol. *1*, reports2047. 10.1186/gb-2000-1-2-reports2047.

6. Jalali-Yazdi, F., Chowdhury, S., Yoshioka, C., and Gouaux, E. (2018). Mechanisms for Zinc and Proton Inhibition of the GluN1/GluN2A NMDA Receptor. Cell *175*, 1520-1532.e15. 10.1016/j.cell.2018.10.043.

7. Wang, H., Lv, S., Stroebel, D., Zhang, J., Pan, Y., Huang, X., Zhang, X., Paoletti, P., and Zhu, S. (2021). Gating mechanism and a modulatory niche of human GluN1-GluN2A NMDA receptors. Neuron *109*, 2443-2456.e5. 10.1016/j.neuron.2021.05.031.

8. Zhang, J.-B., Chang, S., Xu, P., Miao, M., Wu, H., Zhang, Y., Zhang, T., Wang, H., Zhang, J., Xie, C., et al. (2018). Structural Basis of the Proton Sensitivity of Human GluN1-GluN2A NMDA Receptors. Cell Rep. *25*, 3582-3590.e4. 10.1016/j.celrep.2018.11.071.

9. Tajima, N., Karakas, E., Grant, T., Simorowski, N., Diaz-Avalos, R., Grigorieff, N., and Furukawa, H. (2016). Activation of NMDA receptors and the mechanism of inhibition by ifenprodil. Nature *534*, 63–68. 10.1038/nature17679.

10. Chou, T.-H., Tajima, N., Romero-Hernandez, A., and Furukawa, H. (2020). Structural Basis of Functional Transitions in Mammalian NMDA Receptors. Cell *182*, 357-371.e13. 10.1016/j.cell.2020.05.052.

11. Tajima, N., Simorowski, N., Yovanno, R.A., Regan, M.C., Michalski, K., Gómez, R., Lau, A.Y., and Furukawa, H. (2022). Development and characterization of functional antibodies targeting NMDA receptors. Nat. Commun. *13*, 923. 10.1038/s41467-022-28559-3.

12. Zhu, S., Stein, R.A., Yoshioka, C., Lee, C.-H., Goehring, A., Mchaourab, H.S., and Gouaux, E. (2016). Mechanism of NMDA Receptor Inhibition and Activation. Cell *165*, 704–714. 10.1016/j.cell.2016.03.028.

13. Chou, T.-H., Epstein, M., Michalski, K., Fine, E., Biggin, P.C., and Furukawa, H. (2022). Structural insights into binding of therapeutic channel blockers in NMDA receptors. Nat. Struct. Mol. Biol. *29*, 507–518. 10.1038/s41594-022-00772-0.

14. Karakas, E., and Furukawa, H. (2014). Crystal structure of a heterotetrameric NMDA receptor ion channel. Science *344*, 992–997. 10.1126/science.1251915.

15. Zhang, J., Zhang, M., Wang, Q., Wen, H., Liu, Z., Wang, F., Wang, Y., Yao, F., Song, N., Kou, Z., et al. (2023). Distinct structure and gating mechanism in diverse NMDA receptors with GluN2C and GluN2D subunits. Nat. Struct. Mol. Biol. *30*, 629–639. 10.1038/s41594-023-00959-z.

16. Chou, T.-H., Kang, H., Simorowski, N., Traynelis, S.F., and Furukawa, H. (2022). Structural insights into assembly and function of GluN1-2C, GluN1-2A-2C, and GluN1-2D NMDARs. Mol. Cell *82*, 4548-4563.e4. 10.1016/j.molcel.2022.10.008.
